# POSS Polyimide Composite Sealed Triple‐Junction GaAs Thin‐Film Solar Cell for Long‐Term Low Earth Orbit Serve

**DOI:** 10.1002/advs.202516383

**Published:** 2025-12-15

**Authors:** Min Qian, Min Wu, Xiaoyang Xuan, Yang Gao

**Affiliations:** ^1^ School of Physics East China University of Science and Technology Shanghai 200237 P. R. China; ^2^ State Key Laboratory of Space Power‐sources Technology Shanghai Institute of Space Power‐Sources Shanghai 200245 P. R. China; ^3^ College of Chemistry and Chemical Engineering Taishan University Taian Shandong 271000 P. R. China; ^4^ School of Mechanical and Power Engineering Shanghai Key Laboratory of Intelligent Sensing and Detection Technology East China University of Science and Technology Shanghai 200237 P. R. China

**Keywords:** atomic oxygen, POSS polyimide, transmittance, triple‐junction GaAs thin‐film solar cell, ultraviolet

## Abstract

Polyhedral oligomeric silsesquioxane (POSS) polyimide is promising for sealing flexible photoelectronic devices for space applications. However, atomic oxygen interaction with POSS polyimide results in a porous SiO_x_ passivating layer, ultraviolet interaction results in bonding degradation, both causing the transmittance decrease in the visible light range. In this study, the atomic oxygen exposure‐induced transmittance decrease of POSS polyimide is explained and simulated by Rayleigh scattering. Ultrathin oxide films and ultraviolet absorbent are introduced to POSS polyimide by surface‐ and bulk‐phase modifications to improve atomic oxygen and ultraviolet resistance, which achieves ≈0 wt% mass loss upon an eight‐year long‐term atomic oxygen exposure and is deduced by molecular dynamics. The atomic oxygen exposure effect on the sheet resistance of flexible conductive indium tin oxide‐POSS polyimide is explained by band structure calculation. The SiO_2_‐POSS polyimide sealed triple‐junction GaAs thin‐film solar cell exhibits beginning of life (BOL) and end of life (EOL) efficiencies of 27.67% and 23.38% upon an eight‐year long‐term to atomic oxygen. The atomic oxygen reactions with polyimide‐based films are explained by zero‐ and first‐order reactions, and predictive formulas are created for the film mass loss and sealed solar cell performance under the long‐term atomic oxygen exposure. This study suggests a POSS polyimide composite as a packaging film for flexible photoelectronic devices in low Earth orbit.

## Graphical abstract

Ultrathin oxide layer and ultraviolet absorbent are introduced into polyhedral oligomeric silsesquioxane (POSS) polyimide to improve the transmittance conservation and realize near zero mass loss against ultraviolet and atomic oxygen exposures. The POSS polyimide composite sealed triple‐junction GaAs thin‐film solar cell exhibits enhanced endurance of atomic oxygen and thermal cycling for long‐term low Earth orbit service.

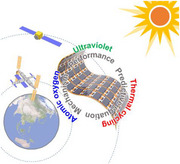

## 1 Introduction

In low Earth orbit, atomic oxygen, ultraviolet, and thermal cycling are major concerns. Atomic oxygen causes surface oxidation and results in mass loss of polymers, with a 5 eV translational energy and a density of ≈10^9^ atoms cm^−3^.^[^ ^1^, ^2^, ^3^, ^4^, ^5^, ^6^, ^7^, ^8^, ^9^, ^10^, ^11^, ^12^ ^]^ Ultraviolet causes chemical bond degradation of materials and as well as accelerates the atomic oxygen erosion, with a dose of 1200 ESH year^−1^.^[^ ^13^, ^14^, ^15^, ^16^ ^]^ Thermal cycling is with a temperature range −40 °C–50 °C at 96 min cycle^−1^.^[^ ^17^, ^18^, ^19^ ^]^ GaAs is a man‐made semiconductor with a direct bandgap of 1.42 eV, a strong absorbance coefficient of 10^4^–10^5^ cm^−1^ in visible and near‐infrared wavelength, and a high carrier mobility of 8500 cm^2^ V^−1^ s^−1^, which is ideal for solar cells.^[^ ^20^, ^21^, ^22^, ^23^, ^24^, ^25^, ^26^, ^27^ ^]^ The conduction band and valence band of GaAs are mainly related to the hybridization of atomic orbitals of arsenic and gallium atoms, where the As 4s electrons mainly form the top of the valence band, while the Ga 4s electrons and the As 4p electrons mainly form the bottom of the conduction band.^[^ ^28^, ^29^, ^30^, ^31^, ^32^, ^33^ ^]^ The symmetric and cubic crystal (FCC) structure and the covalent chemical bonds of GaAs enable its good durability with fewer lattice defects upon harsh space irradiations (Figure S1, Supporting Information crystal structures). Recently, triple‐junction GaAs thin‐film solar cell was produced with an average efficiency of 30% (AM0), which consists of III‐V semiconductors GaInP, GaAs, InGaAs with direct bandgaps of 1.90, 1.42, 1.00 eV.^[^ ^34^ ^]^ The ≈70 µm‐thick flexible triple‐junction GaAs thin‐film solar cell is naturally slightly curving, with a thermal expansion coefficient of 6.5 ppm °C^−1^ (Figure S2, Supporting Information, cell structure).^[^ ^35^, ^36^ ^]^ Transparent POSS polyimide is promising for sealing flexible photoelectronic devices in low Earth orbit, as it exhibits a durable thermal stability within −100 °C–250 °C, a small thermal expansion coefficient of 40 ppm °C^−1^, a durable mechanical tensile strength of 120 MPa and an elongation at break of ≈10%, an ultrasmall bending radius of ≈0 mm (foldable), a high transmittance of 90% in 400–1800 nm, a low dielectric constant of ≈2, and a decreased erosion yield with increasing atomic oxygen fluence with a typical value of 0.12 × 10^−24^ cm^3^ atom^−1^ at a fluence of 4.1 × 10^21^ O atoms cm^−2^ (two‐year dose in low Earth orbit).^[^ ^34^ ^]^ However, atomic oxygen attack to polyhedral oligomeric silsesquioxane (POSS) polyimide forms the porous SiO_x_ passivation layer on top, ultraviolet causes bond breaking, both resulting in degradation of film transmittance.

The relationship between absorbance, transmittance, and reflectance of light is shown in Equation (1).

(1) $$I_{0}=I_{a}+I_{r}+I_{t}$$

where the *I*_0_ is the intensity of incident light, *I_a_* is the intensity of absorbed light, *I_r_* is the intensity of reflected light, *I_t_* is the intensity of transmitted light.

**Figure 1:**
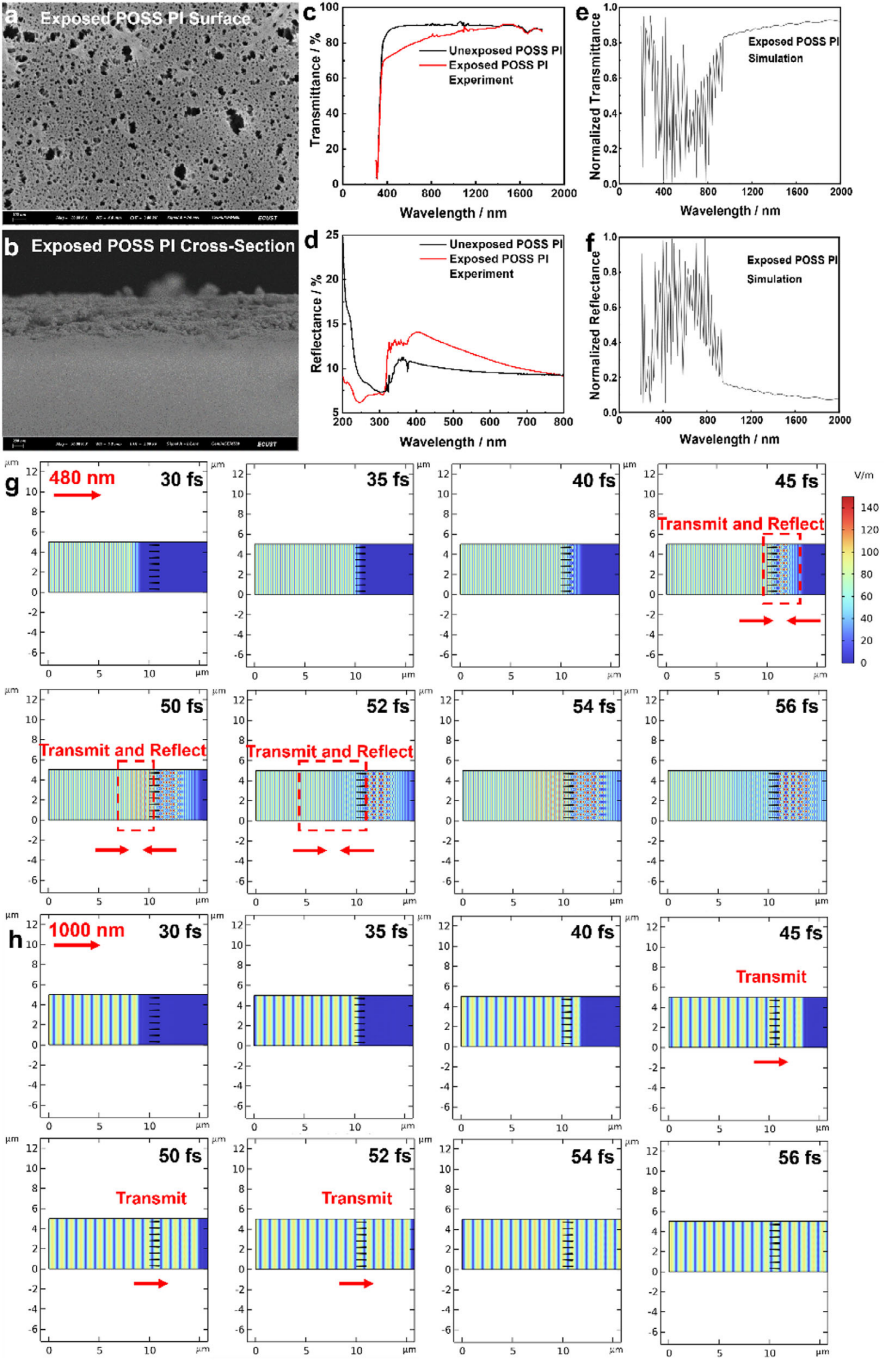
Rayleigh scattering of porous SiO_x_ grid layer. a) Surface and b) cross‐section SEM images of POSS polyimide films after a 2.64 × 10^20^ O atoms cm^−2^ atomic oxygen exposure. c) Transmittance and d) reflectance of POSS polyimide films before and after the atomic oxygen exposure (experimental data). e) Transmittance and f) reflectance of atomic oxygen exposed POSS polyimide films (simulation data). The simulated propagation trajectories of g) a 480 nm and h) a 1000 nm light in a porous SiO_x_ grid layer.

Atomic oxygen interaction with polyimide products, volatile gases, resulting in the mass loss with a constant erosion yield of 3.0 × 10^−24^ cm^3^ O atom^−1^ (Equation 2).^[^ ^37^, ^38^, ^39^, ^40^, ^41^, ^42^, ^43^ ^]^ POSS polyimide products, volatile gases, and non‐volatile porous SiO_x_ network (Equation 3).^[^ ^34^, ^37^, ^38^, ^39^, ^40^, ^41^ ^]^ The non‐volatile Si_7_O_9_ POSS cage left on the surface oxidizes toward SiO_2_ and connects with each other to form a porous network, resulting in a transmittance decrease in the visible range.^[^ ^34^, ^37^, ^38^, ^39^, ^40^, ^41^ ^]^ The transmittance and reflectance of POSS polyimide both decrease after atomic oxygen exposure, and the absorbance increases.^[^ ^34^, ^37^, ^41^ ^]^ This is because part of the incident light scatters rather than transmits in the porous SiO_x_ layer (grid) according to the Rayleigh scattering. The particle size (rib width of SiO_x_ grid) is around tens of nanometers. Thus, photon with smaller wavelength around a few hundred of nanometers (**Figure** **1**a,b) are prone to be scattered, and thus the transmittance decreases while absorption increases in the visible light range (Figure 1c,d).^[^ ^41^ ^]^ To inhibit the Rayleigh scattering, ultrathin oxide layers are designed in this study to prevent the formation of porous SiO_x_ layers.

(2) $$x\;O+y\;Polyimide\to CO_{2}↑+CO↑+H_{2}O↑+NO↑$$

(3) $$\begin{array}{ccc} x\;O+y\;POSS\;Polyimide\to CO_{2}↑ & + & CO↑+H_{2}O↑\\ & & +NO↑+SiO_{2}↓ \end{array}$$

Ultraviolet in space, with a wavelength range between 100 and 400 nm, exhibits high photon energy (E = hν, where E is the photon energy in J, h is the Planck constant of 6.626 × 10^−34^ J·s, ν is the light frequency in Hz) in the range 3.1–12.4 eV. A high‐energy photon is able to photo‐dissociate molecules. This process generally involves the molecule (or its electrons) first resonantly absorbing a photon, reaching an excited state. This excited state either falls directly to the dissociated state or has a strong coupling with an energy‐equivalent dissociated state, after which the molecule proceeds along the dissociation path. Table S1 (Supporting Information) shows the typical bonding energy of single and double bonds in polyimide, as the ultraviolet photon energy is much higher than the single chemical bonding energy in polymer, long‐term ultraviolet irradiation causes severe degradation of single bonds (the phenyl groups), as demonstrated by the Fourier transform infrared spectroscopy spectra (FTIR) (Table S2, Supporting Information).^[^ ^34^ ^]^ The chemical bonds are made of electron clouds, which also determine the HOMO‐LUMO energy gap as well as the cutoff wavelength of transmittance (Figure S3, Supporting Information). Ultraviolet irradiation on polyimide causes bond degradation and distortion of electron clouds, resulting in transmittance decrease (absorption increase) in the visible light range.^[^ ^34^ ^]^ To improve the material transmittance durability to ultraviolet, 5 wt% nano CeO_2_ is reinforced to space antiradiation glass as an ultraviolet absorbent. The photoexcitation causes the CeO_2_ to transition to a higher electronic state (Ce^4+^ to Ce^3+^), which then releases energy (photons at visible range) back to a lower energy level after a relaxation process (Ce^3+^ to Ce^4+^).^[^ ^44^, ^45^ ^]^ As polyimide is an organic material, organic ultraviolet absorbent is explored in this study according to the “like dissolves like” principle, to improve the polyimide chemical bond stability and enhance the transmittance durability upon ultraviolet radiation.

To enhance the transparent POSS polyimide endurance to atomic oxygen and ultraviolet, ultrathin oxide layers and ultraviolet absorbent materials are introduced in this study. Film samples for atomic oxygen exposure in this study in shown in **Table** **1** (Figure S4, Supporting Information). The chemical reaction of atomic oxygen interaction with polyimide‐based materials is explained by zero‐ and first‐order reactions, predictive equations, and molecular dynamic simulations. POSS polyimide composite‐based conductive films and sealed triple‐junction GaAs thin‐film solar cell are explored, and their performance evolutions with atomic oxygen exposure are deduced by band structure calculations and predictive equations. Atomic oxygen interaction on POSS polyimides in prior studies results in a porous SiO_x_ surface, a decreased transmittance, and a mass loss. To explain this phenomenon, a finite element analysis simulation using COMSOL software has been performed to explain the reason for the transmittance decrease is because the Rayleigh scattering occurs at the porous SiO_x_ microstructure. To solve this problem, ultrathin oxide layers have been introduced in this work, including SiO_2_, Al_2_O_3_, and ITO. Sputtering and atomic‐layer deposition are both applied for depositing 50 nm thick SiO_2_ layers, and achieving a ∼zero mass loss upon a 1.6 × 10^22^ O atoms cm^−2^ atomic oxygen exposure.

**Table 1:** Details of the film samples in this study.

Label	Details
PI	polyimide
POSS PI	POSS polyimide
SP‐SiO_2_	50 nm‐thick sputtered SiO_2_ on POSS polyimide
SP‐SiO_2_‐UV	50 nm‐thick sputtered SiO_2_ on POSS polyimide with 2 wt% UV absorbent
ALD‐SiO_2_‐UV	50 nm‐thick atomic‐layer‐deposited SiO_2_ on POSS polyimide with 2 wt% UV absorbent
ALD‐Al_2_O_3_	50 nm‐thick atomic‐layer‐deposited Al_2_O_3_ on POSS polyimide
SP‐ITO10‐UV	10 nm‐thick sputtered ITO on POSS polyimide with 2 wt% UV absorbent
SP‐ITO20	20 nm‐thick sputtered ITO on POSS polyimide
SP‐ITO50‐UV	50 nm‐thick sputtered ITO on POSS polyimide with 2 wt% UV absorbent

## 2 Results and Discussion

### 2.1 Rayleigh Scattering of Porous SiOx layer

The 7.3 wt% Si_7_O_9_ POSS cages in polyimide left on the surface act as isolate nucleation sites. During atomic oxygen exposure, the 7.3 wt% Si_7_O_9_ POSS cages are oxidized toward SiO_2_, and connect with each other forming the SiO_x_ passivating layer. The SiO_x_ passivating layer exhibits network structures as SiO_x_ grid, with rib width at the tens of nanometer scale (Figure 1a,b). The SiO_x_ ribs act as particles, and the size (tens of nanometer) is much smaller than that of the incident light wavelength (hundreds of nanometer), thus the Rayleigh scattering occurs. As the SiO_x_ grid layer introduces a Rayleigh scattering to the incident light, the scattering intensity is stronger at higher frequency range, resulting in a lower transmittance and a higher reflectance at the visible range than the infrared range (Figure 1c,d). The Rayleigh scattering of the SiO_x_ grid layer on POSS polyimide surface is also simulated by the COMSOL Multiphysics software, indicating the same trend (Figure 1e,f). According to the Rayleigh scattering, when the SiO_x_ rib particle size is much smaller (one tenth) than the incident light wavelength, the photon scattering intensity of visible light is much stronger than that of infrared light.^[^ ^46^, ^47^, ^48^, ^49^ ^]^ This is observed by experiment (Figure 1c,d), as well as by simulation (Figure 1e). From the simulation (Figure 1f), within the 300–500 nm range, reflectance exhibits a fluctuating upward trend, while showing a fluctuating downward trend between 500 and 900 nm. This occurs because within this spectral range, the dimensions of microstructures and their gaps are ≈0.1–1 times of the wavelength. At this scale, Rayleigh scattering occurs where surface microstructures interact with light, enhancing reflection.^[^ ^48^ ^]^ Under longer wavelengths such as 1000 nm, reflectance fluctuations diminish as the microstructure size becomes substantially smaller than the wavelength, weakening light‐microstructure interactions. This phenomenon explains the slight reflectance increase at 400 nm after atomic oxygen bombardment (Figure 1d). The cross‐section structure of the SiO_x_ passivating layer is modeled using the Comsol Multiphysics software (Figure 1g,h; Videos S1 and S2, Supporting Information). When a 480 nm light hits the porous SiO_x_ layer, the SiO_x_ grid structure and light wave interact, and some photons reflect at 45 fs (Figure 1g). When a 1000 nm light hits, most photons transmit (Figure 1h). To improve the transmittance of atomic oxygen exposed POSS polyimide, ultrathin oxide layers are applied on the surface to prevent the formation of porous SiO_x_ layer. To dynamically analyze the light‐microstructure interactions, time‐domain simulations were performed using 480 nm and 1000 nm wavelengths (intensity: 100 V m^−1^) (Videos S1 and S2, Supporting Information). The case of 480 nm is analyzed as follows. 1) At 30 fs: Light propagates freely ahead of microstructures. 2) At 35 fs: Light traverses the microstructure region. The 480 nm wave impinges obliquely, generating backward‐propagating reflected waves that interfere with forward‐propagating waves. 3) At 40 fs: The wavefront crosses the microstructure region (1 µm), extending the interference zone. 4) At 45 fs: Clear spherical wave interference patterns emerge behind microstructures, indicating structural modulation meeting optical coherence requirements. 5) At 50 fs: Interference emerges at ≈4–5 µm, distinct from the planar wave profile at 30 fs. The interference region expands from ≈5 to ≈1 µm, confirming reflection sources from microstructures. 6) The periodic variation in the intensity of the reflected light field indicates that, due to the microscopic structure, part of the energy is reflected. Conversely, at 1000 nm, light propagates uniformly through the microstructure region without obstruction or significant reflection, further confirming enhanced reflection at shorter wavelengths and negligible interaction at longer wavelengths.

### 2.2 Atomic Oxygen Reaction Mechanism and Predictive Models

**Figure 2:**
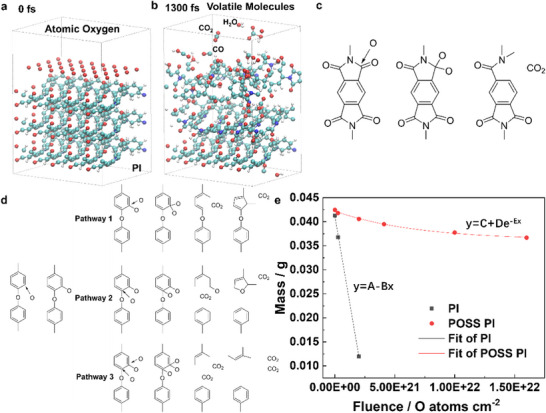
a,b) Molecular dynamic simulations of atomic oxygen interaction with polyimide. c,d) Bond evolutions of volatile CO_2_ formation upon atomic oxygen interaction with polyimide. e) Predictive equations of atomic oxygen interaction with polyimide and POSS polyimide.

Both space and laboratory atomic oxygen‐polyimide gas‐surface scattering experiments in the 1990s show the volatile reaction products of CO and CO_2_.^[^ ^50^ ^]^ **Figure** **2**a,b illustrates the molecular dynamic simulation of the atomic oxygen interaction with polyimide, producing volatile molecules of CO, CO_2_, and H_2_O at 1300 fs (Video S3, Supporting Information). The atomic oxygen matrix is designed with comparable density in space (≈10^9^ cm^−3^), and with an incident velocity of 7.9 km s^−1^. After bombardment by atomic oxygen arrays, the C─C bonds on the polyimide surface fractures, forming stable long‐chain C─O─H structures adsorbed on subsurface molecular layers.^[^ ^51^, ^52^ ^]^ Most detached H atoms volatilize as atomic hydrogen due to spatial separation, while a minority “luckily” combines with O or H atoms to form H_2_O or H_2_. Some O atoms react with C to generate volatile CO, CO_2_, and unstable small molecules (e.g., CHO_3_, CO_2_H) (Figure 2c,d). The atomic oxygen interaction with polyimide is presented as follows (Video S3, Supporting Information). 1) 0–50 fs: Atomic oxygen reaches the polyimide surface, and displaces the outermost H atoms on carbon chains via exchange reactions. C─H bonds convert to C─O bonds, with most H volatilizing as atoms and minor H_2_ formation. 2) 50–100 fs: Some rebound oxygen atoms lacking bonding sites combine with H to form volatile H_2_O. 3) >100 fs: Carbon chains fracture, releasing volatile CO or CO_2_. There are two carbon sources. One is the C atoms near ─C─N─C─O‐ bonds in nitrogenous five‐membered rings, and other is the C atoms near ─O─C─C─ bonds in non‐nitrogenous six‐membered rings. The mechanism of the five‐membered ring evolution is further presented as follows (Figure 2c). During 0–50 fs, the bombardment increases reactive oxygen near these regions, forming ─C─N⋯O─C─O─ and ─O─C─C─O intermediates. At 50–100 fs, the five‐membered ring─C─N… combines with other C atoms and reforms C─N─C bond. The five‐membered ring structure disappears, and the O‐C‐O structure is independent and volatilizes as CO_2_. For the ─O─C─C─O moiety on the six‐membered ring, the evolution of the two carbon atoms follows divergent pathways (Figure 2d). 1) Pathway 1: One C atom bonds with ambient atomic oxygen, forming ─O─C⋯O─C─O─. The O─C─O segment detaches and volatilizes as CO_2_, while the residual ─O─C⋯ reconnects to a carbon atom from the original six‐membered ring, forming a new ─O─C─C─ bond. This collapses the six‐membered ring into a five‐membered carbocycle. 2) Pathway 2: Another C atom bonds with ambient atomic oxygen. And this C and the O, which bridges the two six‐membered rings together form a ─O─C─O─ structure. This unit detaches as volatile CO_2_. The terminal oxygen on the opened chain subsequently reorganizes into an oxygen‐containing five‐membered ring. 3) Pathway 3 (Combination of Pathways 1 and 2): Both oxygen‐linked carbon atoms react with ambient atomic oxygen, volatilizing as two CO_2_ molecules. The residual carbon skeleton forms an open‐chain structure.

The atomic oxygen interactions with polyimide and POSS polyimide are explained by zeroth‐order (Equation 2) and first‐order reactions (Equation 3), respectively. From Equation 2, the reagent concentrations of atomic oxygen and polyimide are unchanged, resulting in a constant reaction speed.  From the fitting of experiment data (Figure 2e), the mass loss of polyimide versus atomic oxygen fluence is predicted by Equation 4, where the reaction speed of atomic oxygen interaction with polyimide is constant 3.0 × 10^−24^ cm^3^ O atom^−1^.

(4) y=A−B·x

where y is the final mass of the reactant polyimide in g, *A* is the initial mass of the reactant polyimide in g, *B* is the mass loss rate caused by a 1 cm^2^ of oxygen atoms in 1.455 × 10^−23^ g/O atoms cm^−2^, x is the atomic oxygen fluence in O atoms cm^−2^.

From Equation 3, the reagent concentrations of exposed POSS polyimide decrease during the atomic oxygen exposure as the gradual formation of SiO_x_ on surface, where the POSS polyimide resulting in a decreasing reaction speed.  From the fitting of experiment data (Figure 2e), the mass loss of POSS polyimide versus atomic oxygen fluence is predicted by Equation 5, where the reaction speed of atomic oxygen interaction with POSS polyimide decreases with increasing atomic oxygen fluence, with 0.16 × 10^−24^ cm^3^ O atom^−1^ at 2.0 × 10^21^ O atoms cm^−2^, 0.12 × 10^−24^ cm^3^ O atom^−1^ at 4.0 × 10^21^ O atoms cm^−2^, 0.8 × 10^−25^ cm^3^ O atom^−1^ at 1.0 × 10^22^ O atoms cm^−2^, 0.6 × 10^−25^ cm^3^ O atom^−1^ at 1.6 × 10^22^ O atoms cm^−2^.

(5) $$y=C+D\cdot e^{-Ex}$$

where y is the final mass of the reactant POSS polyimide in g, *C* + *D* is the initial mass of the reactant POSS polyimide in g, *D* is the maximum mass loss in g, *E* is reaction speed constant in cm^2^ O atom^−1^, x is the atomic oxygen fluence in O atoms cm^−2^.

### 2.3 Ultraviolet Absorbent Bulk‐Phase Reinforcement

**Figure 3:**
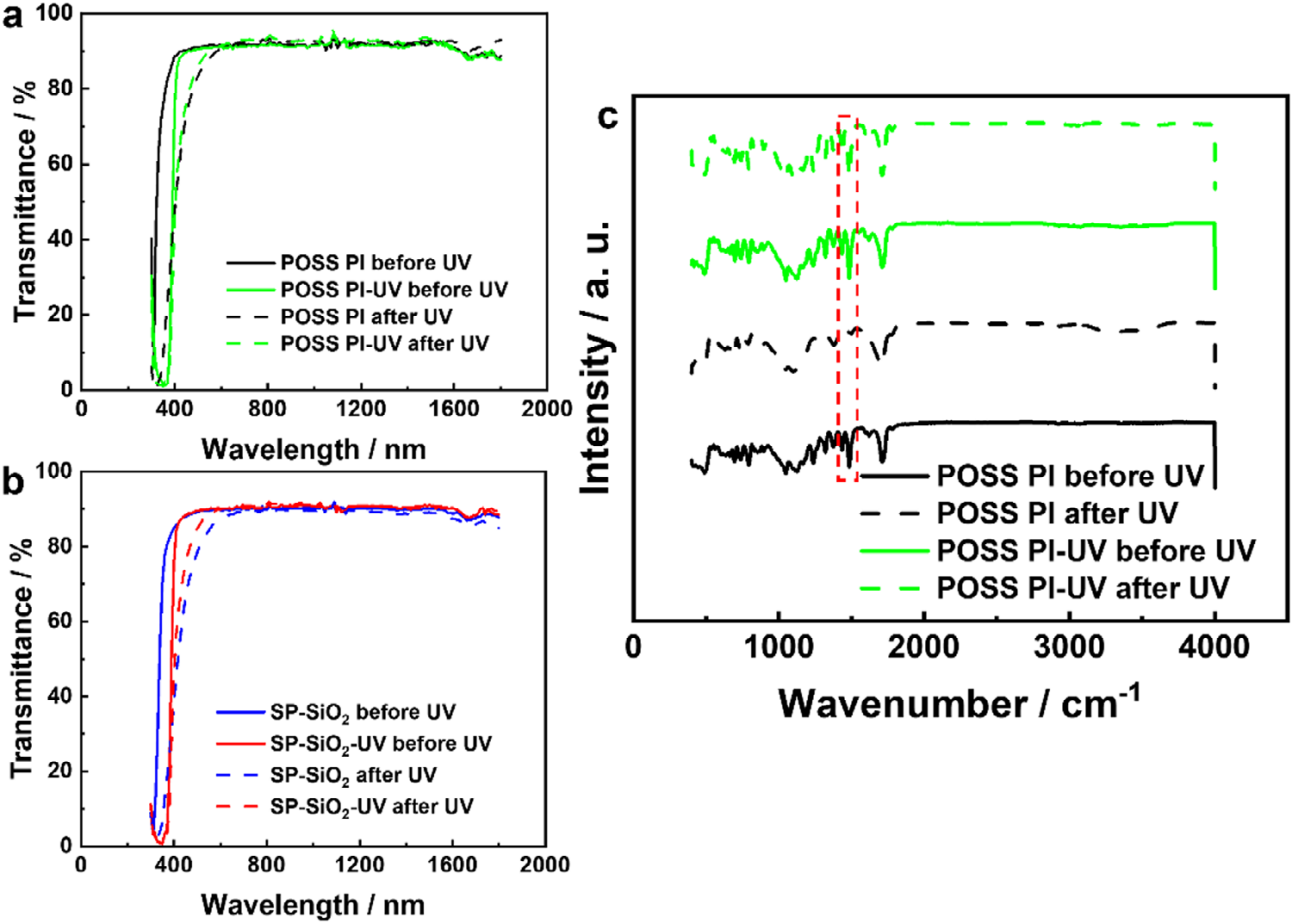
Transmittance and FTIR spectra. a,b) Transmittance of POSS and SP‐SiO_2_‐POSS polyimides, and c) FTIR spectra of POSS polyimides, with and without ultraviolet absorbent before and after ultraviolet exposure.

The samples with ultraviolet absorbent (POSS PI‐UV and SP‐SiO_2_‐UV) show an increased cutoff wavelength at ≈380 nm compared to the samples without ultraviolet absorbent (POSS PI and POSS‐SiO_2_), and this is how the 2 wt% ultraviolet absorbent works. Ultraviolet absorbents protect materials from ultraviolet damage by effectively absorbing ultraviolet light (with wavelength <380 nm) and converting it into heat or fluorescence. The higher the absorption is, the lower the transmittance is (Equation 1). Ultraviolet absorbents delay discoloration and yellowing in polymers caused by photo‐dissociation and photo‐oxidation reactions while maintaining transparency in the visible light range. The 2 wt% ultraviolet absorbent bulk‐phase reinforcement is to improve the light stability of polyimide, as it effectively absorbs ultraviolet light of 270–380 nm nanometers and hardly absorbs visible light, which protects the polyimide from absorbing ultraviolet and degrading.^[^ ^16^ ^]^ The films with the 2 wt% ultraviolet absorbent additives, show smaller the transmittance drop with wavelength higher than 400 nm (**Figure** **3**a,b and **Table** **2**). The POSS polyimides with ultraviolet absorbent (POSS PI‐UV) exhibit better transmittance conservation than the POSS polyimides without ultraviolet absorbent (POSS PI) (Figure 3 and Table 2). At 550 nm, the POSS PI‐UV exhibits only a 0.90% transmittance drop after the 150 ESH ultraviolet exposure, while the POSS PI exhibits a 3.50% transmittance drop (Figure 3a). At 550 nm, the SP‐SiO_2_‐UV exhibits a 1.54% transmittance drop after the 150 ESH ultraviolet exposure, while the SP‐SiO_2_ exhibits a 5.38% transmittance drop (Figure 3b). FTIR shows that with the ultraviolet absorbent, the chemical bonds are well conserved after the ultraviolet exposure, while without the ultraviolet absorbent, the FTIR peak at 1485 cm^−1^ disappears, indicating the phenyl ring degradation (Figure 3c; Table S2, Supporting Information). The ultraviolet absorbent has a more than 10 wt% solubility in the solvent. The 2 wt% addition of ultraviolet absorbent is compatible with the polyimide, and it effectively absorbs ultraviolet light of 270–380 nm and hardly absorbs visible light, which is especially suitable for transparent polymers (Figure 3a,b).^[^ ^16^ ^]^ The thermal decomposition temperature and mechanical properties are well conserved, with 460 °C and 115 MPa for the POSS PI, 465 °C and 105 MPa for the POSS PI‐UV (Figure S5, Supporting Information). The polyimides with and without POSS reinforcement show similar transmittance after the ultraviolet exposure, indicating the POSS introduced to the polyimide in this study does not show a detectable improvement in ultraviolet protection (Figure S6a, Supporting Information). The ultraviolet‐induced transmittance decrease comes from the light absorption of the chemical bond evolution. The transmittance ($\int_{300\;nm}^{1800\;nm}Transmittance\;d\lambda$) of POSS polyimides decrease by 4.79% and 11.12% after 150 and 2500 ESH ultraviolet exposure, respectively (Figure S6b,c, Supporting Information). Although the curve in Figure S6c (Supporting Information) is lack of test points, it presents a nonlinear trend based on the current two test points. As there is not a simple and direct mathematical relationship between the ultraviolet‐induced chemical bond evolution and polyimide transmittance loss. It is worth conducting a systematic experimental investigation with sufficient test points along with the ultraviolet dose in further work.

**Table 2:** Transmittance data at 550 nm. a) POSS and b) SP‐SiO_2_‐POSS polyimides with and without ultraviolet absorbent before and after ultraviolet exposure.

Samples	Transmittance @ 450 nm / %		Transmittance @ 500 nm / %		Transmittance @ 550 nm / %	
	Before UV	Before UV	Before UV	After UV	Before UV	After UV
POSS PI	90.18	73.07	91.11	83.37	91.74	88.24
POSS PI‐UV	89.56	76.21	90.52	85.97	90.88	89.98
POSS PI‐SiO_2_	87.98	66.30	89.11	78.44	89.61	84.24
POSS PI‐SiO_2_‐UV	88.34	75.18	89.65	84.80	89.92	88.38

### 2.4 Ultrathin Oxide Layer Surface Reinforcement

**Figure 4:**
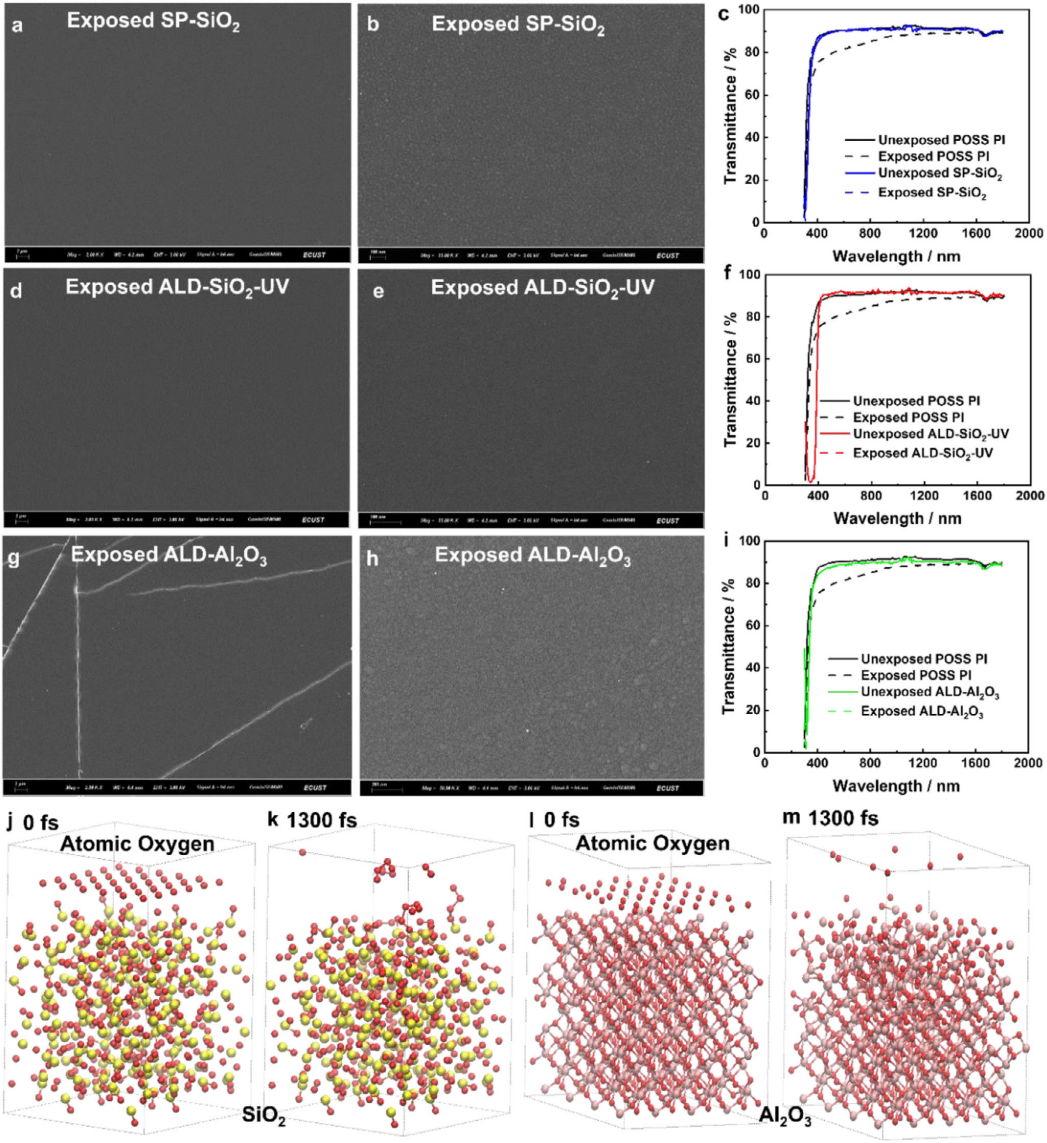
Oxide layer/POSS polyimide composites with enhanced atomic oxygen durability. a) Low‐, b) high‐magnification SEM images, c) transmittance of SP‐SiO_2_ POSS polyimide. d) Low‐, e) high‐magnification SEM images, f) transmittance of ALD‐SiO_2_‐UV POSS polyimide. g) Low‐, h) high‐magnification SEM images, i) transmittance of ALD‐Al_2_O_3_ POSS polyimide, upon a 2.64 × 10^20^ O atoms cm^−2^ atomic oxygen exposure. Molecular dynamic simulations of j,k) SP‐SiO_2_, l,m) Al_2_O_3_ films upon atomic oxygen exposure.

Sputtering and atomic‐layer deposition are utilized to depositing ultrathin oxide layer on top of POSS polyimides. The surface morphology of SP‐SiO_2_ and ALD‐SiO_2_‐UV keep smooth after the atomic oxygen, as shown in their low‐ and high‐magnification SEM images (**Figures** **4**a,b,d,e). The transmittance data of SP‐SiO_2_ and ALD‐SiO_2_‐UV exhibit no degradation at all (Figure 4c,f), indicating a complete conservation of the 90% transmittance in the range 400–1800 nm, in comparison with the results of control POSS polyimide (Figure 1c,d). The erosion yields of POSS polyimide and polyimide films are derived from etch depth by Equation 6. The erosion yields of ultrathin oxide/POSS polyimide films are derived from mass loss by Equation 7.

(6) $$E_{y}=d/F$$

(7) $$E_{y}=\Delta m/A\rho F$$

where *Ey* is erosion yield in cm^3^ O atom^−1^, *d* is etch depth in cm, *ρ* is material density of 1.41 g cm^−3^, *A* is exposed area of cm^2^, *F* is atomic oxygen fluence in atoms cm^−2^.

The erosion yields of control PI, POSS PI, SP‐SiO_2_, SP‐SiO_2_‐UV, and ALD‐SiO_2_‐UV are 3.18 ± 0.11, 0.19 ± 0.03, 0.36 ± 0.13, 0.00 ± 0.16, −0.01 ± 0.13 × 10^−24^ cm^3^ O atom^−1^, respectively (**Tables** **3** and **4**). The bulk‐phase POSS doping improves the atomic oxygen resistance of polyimides by a factor of 16.74. At the atomic oxygen fluence of 2.64 × 10^20^ O atoms cm^−2^, the erosion yields of POSS PI and SP‐SiO_2_ are comparable. The bulk‐phase ultraviolet absorbent doping further improves the atomic oxygen resistance of polyimide by achieving near zero erosion yield of SP‐SiO_2_‐UV and ALD‐SiO_2_‐UV, indicating both sputtering and atomic layer deposition work well for achieving ultrathin oxide films on POSS polyimides. The bulk‐phase ultraviolet absorbent doping further improves the atomic oxygen resistance, because the atomic oxygen facility generates ultraviolet during operating, and the ultraviolet absorbent is a polymer stabilizer. In this way, the 2 wt% bulk‐phase doping of ultraviolet absorbent improves both the atomic oxygen (Table 4) and ultraviolet resistance (Table 2). The transparent ultrathin Al_2_O_3_ layer is deposited on POSS polyimide film by atomic‐layer deposition, with a slightly lower transmittance than that of ultrathin SiO_2_ layer at 400–600 nm (Figures 4c,f,i). The strip boundaries in SEM images are common morphologies in deposited Al_2_O_3_ layer.^[^ ^53^, ^54^ ^]^ The ALD‐Al_2_O_3_‐UV also exhibits no transmittance degradation and an ultralow erosion yield of 0.01 ± 0.12 × 10^−24^ cm^3^ O atom^−1^ after the atomic oxygen exposure (Table 4). Overall, these 50 nm thick ultrathin oxide on POSS polyimides help achieve a full conservation to atomic oxygen with near zero erosion yield and no degradation in transmittance, and as well as maintained the flexibility of POSS polyimide, indicating them promising candidates for space packaging films, while thicker oxide films crack during bending. The atomic oxygen reactions with polyimide and composites are simulated by molecular dynamics (Figures 2a,b and 4j–m; Videos S3–S5, Supporting Information). The high‐speed atomic oxygen interaction with polyimide results in volatile gases (Equation 2), where the O_2_, CO, and H_2_O molecules are easily to observed both by simulation (Figure 2a,b; Video S3, Supporting Information) and gas‐surface scattering experiment.^[^ ^50^ ^]^ The high‐speed atomic oxygen interaction with ultrathin oxide SiO_2_ and Al_2_O_3_ films on POSS polyimides results in the most elastic scattered oxygen atoms and molecules, responding to the zero mass loss. The SiO_2_ and Al_2_O_3_ lattices keep unchanged, while compressive phase transitions occur due to momentum transfer during the atomic oxygen attack, indicating the material resilience (Figure 4j–m; Videos S4,S5, Supporting Information). For POSS PI, the elemental concentrations of Si and O increase significantly after the atomic oxygen exposure, from 7.2 at% Si 2p and 16.7 at% O 1s, to 18.04 at% Si 2p and 43.04 at% O 1s. The atomic ratios of Si 2p and O 1s on POSS PI, SP‐SiO_2_, SP‐SiO_2_‐UV, ALD‐SiO_2_‐UV, ALD‐Al_2_O_3_‐UV surfaces after the atomic oxygen exposure are 1:2.39, 1:2.01, 1:2.06, 1:1.91, and the atomic ratios of Al 2p and O 1s on ALD‐Al_2_O_3_‐UV surface after the atomic oxygen exposure is 2:2.82, indicating a saturated oxidation of the ultrathin oxide layers (**Table** **5**; Figure S7, Supporting Information). We used an 8 in wafer area ALD facility in this study. Recently, a large roll‐to‐roll ALD coating platform is developed with great scalability and manufacturability by combining the large spatial ALD technology with the roll‐to‐roll vacuum technology (width>1.5 m), which may show potential for space deployment feasibility. The quantitative comparison of erosion yield improvements is summarized in **Table** **6**, where the erosion yields have been significantly reduced by introducing ultrathin oxide layers, with ∼zero erosion yield.

**Table 3:** Erosion yield of mashed POSS polyimide and polyimide films derived from etch depth.

Sample	POSS PI	Kapton H	PI
Average Depth / um	0.50 ± 0.09	7.92 ± 0.14	8.39 ± 0.29
Erosion Yield / ×10^−24^ cm^3^ atom^−1^	0.191 ± 0.034		3.176 ± 0.108

**Table 4:** Erosion yield of ultrathin oxide/POSS polyimide composite films derived from mass loss.

Sample	SP‐SiO_2_	ALD‐SiO_2_‐UV	ALD‐Al_2_O_3_	SP‐SiO_2_‐UV
Mass before exposure / mg	8.058 ± 0.020	8.422 ± 0.019	9.162 ± 0.022	10.688 ± 0.030
Mass after exposure / mg	7.971 ± 0.024	8.426 ± 0.025	9.160 ± 0.019	10.688 ± 0.022
Mass loss / mg	0.086 ± 0.032	−0.003 ± 0.031	0.003 ± 0.029	0.000 ± 0.037
Erosion Yield / ×10^−24^ cm^3^ atom^−1^	0.364 ± 0.133	−0.013±0.132	0.011 ± 0.121	0 ± 0.156

**Table 5:** Surface atomic concentrations of polyimide and its composite films upon atomic oxygen exposure.

	Relative atomic concentration / %							
	C 1s	O 1s	Si 2p	N 1s	F 1s	In 3d	Sn 3d5	Al 2p
Unexposed POSS PI	66.9	16.7	7.2	2.8	6.4	0.00	0.00	0.00
Exposed POSS PI	32.78	43.04	18.04	3.24	2.89	0.00	0.00	0.00
Exposed SP‐SiO_2_	24.77	49.16	24.40	1.67	0.00	0.00	0.00	0.00
Exposed SP‐SiO_2_‐UV	16.32	56.32	27.37	0.00	0.00	0.00	0.00	0.00
Exposed ALD‐SiO_2_‐UV	14.93	54.02	28.30	1.45	1.30	0.00	0.00	0.00
Exposed ALD‐Al_2_O_3_	26.02	40.43	0.00	0.00	4.82	0.00	0.00	28.73

**Table 6:** Comparison of erosion yields.

Sample	Erosion yield / cm^3^ atom^−1^	Erosion yield improvement factor relative to Kapton H	Erosion yield improvement factor relative to unmodified PI
POSS PI	0.191 ± 0.034	15.71	16.63
SP‐SiO_2_	0.364 ± 0.133	8.24	8.73
ALD‐SiO_2_	−0.013 ± 0.132	∞	∞
ALD‐Al_2_O_3_	0.011 ± 0.121	272.73	288.73
SP‐SiO_2_‐UV	0 ± 0.156	∞	∞

### 2.5 Flexible Conductive Ultrathin ITO Layer/POSS polyimide

**Figure 5:**
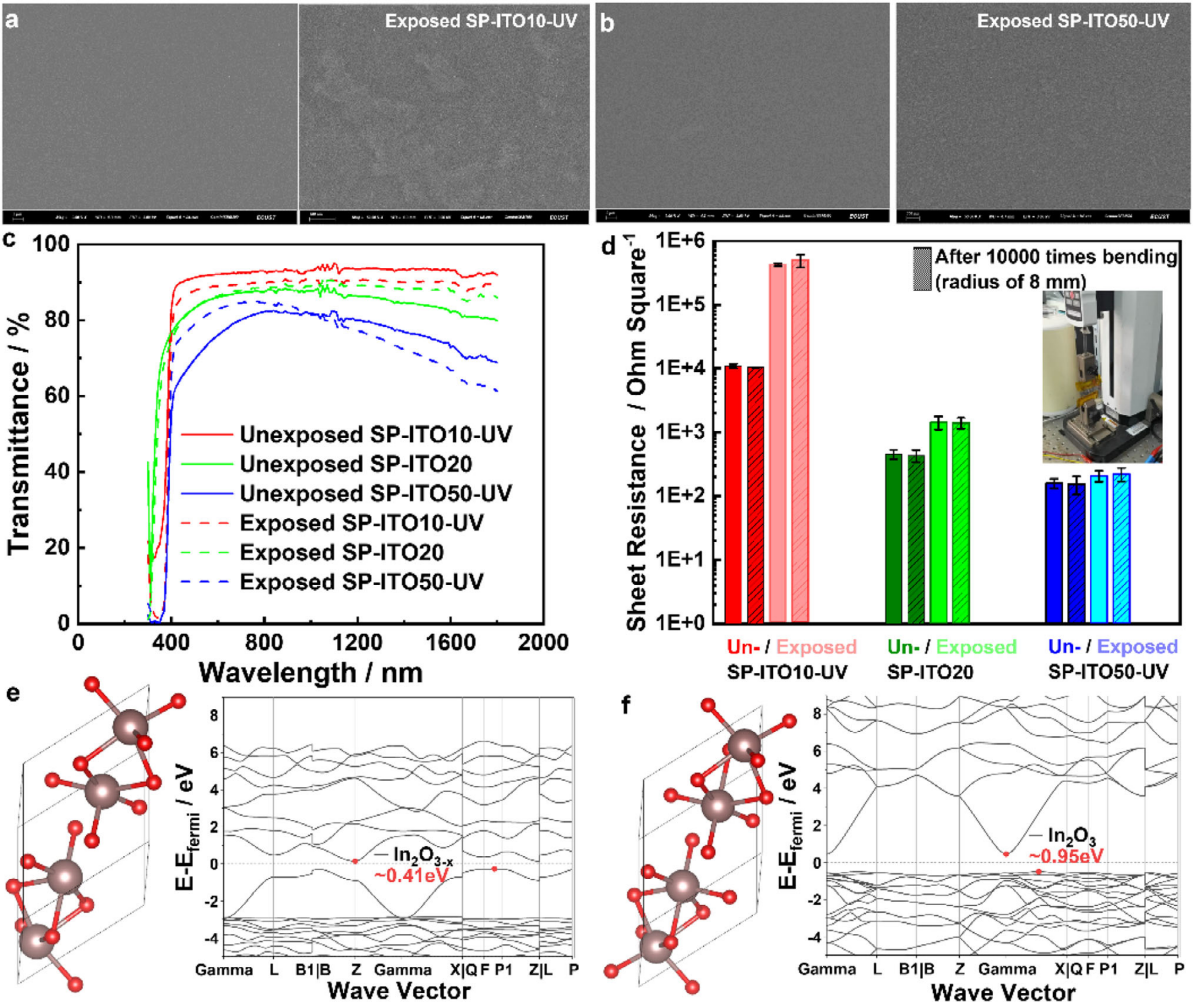
SP‐ITO POSS polyimide conductive films. a.b) Low‐ and high‐magnification SEM images, c) transmittance of SP‐ITO POSS polyimide films upon a 2.64 × 10^20^ O atoms cm^−2^ atomic oxygen exposure. d) Sheet resistances of unexposed and atomic oxygen exposed SP‐ITO POSS polyimide films after 10 000 bending cycling. Band gap evolution of SP‐ITO POSS polyimide films e) before and f) after AO exposure.

The atomic oxygen exposure reaction with ultrathin ITO/POSS polyimides is investigated in terms of the 10, 20, and 50 nm thick ITO layers, respectively.^[^ ^55^, ^56^, ^57^ ^]^ The 10 nm thick ITO layer exhibits isolated ITO islands on surface (**Figure** **5**a), and the 50 nm thick ITO layer exhibits a continuous film (Figure 5b), and both samples show near zero erosion yields upon the atomic oxygen exposure (**Table** **7**). The transmittance of SP‐ITO/POSS polyimide decreases with increasing ITO thickness from 10 nm to 50 nm, and all ITO sample transmittance data remain stable above 80% at 550 nm after the atomic oxygen exposure (Figure 5c). The O 1s concentrations increase by a few percentages in all ITO samples after the atomic oxygen‐induced oxidation reaction (**Table** **8**; Figure S8, Supporting Information). For the 10 nm ITO/POSS polyimide film (SP‐ITO10‐UV), the sheet resistance increases by over two orders of magnitude after the atomic oxygen exposure, from 10 963.33 ± 797.40 to 424600.00 ± 206238.90 Ω^−1^. For 20 nm ITO/POSS polyimide film (SP‐ITO20), the sheet resistance increases by 3 times after the atomic oxygen exposure, from 454.49 ± 79.45 to 1437.33 ± 335.98 Ω □^−1^. For 50 nm ITO/POSS polyimide film (SP‐ITO50‐UV), the sheet resistance keeps relatively stable after the atomic oxygen exposure, from 159.17 ± 27.01 to 207.97 ± 40.66 Ω □^−1^ (Figure 5d and **Table** **9**). One of the reasons is that for the 10 nm ITO/POSS polyimide film (SP‐ITO10‐UV), the ITO domain a few hundred nanometers in size is isolated from each other (Figure 5a), after the atomic oxygen exposure, the unsaturated In ion at edge (Figure 5e) is further oxidized (Figure 5f), which causes the increase in band gap from 0.41 to 0.91 eV, resulting in an increase in the sheet resistance. The sheet resistance durability of ITO/POSS polyimide upon atomic oxygen increase with ITO thickness, where a 50 nm ITO layer is able to conserve the sheet resistance as well as transmittance (Figure 5d and Table 9). A 10000 time bending with a bending radius of 8 mm is conducted to all ITO/POSS polyimide films both before and after the atomic oxygen exposure, all indicating durable bending resistance with stable sheet resistance before and after the bending (Figure 5d and Table 9), indicating that 50 nm thick ultrathin ITO keeps tight adhesion the POSS polyimide, which is promising for serving as transparent, conductive, flexible films or for preventing the static electricity discharge in space.

**Table 7:** Erosion yield of ultrathin ITO/POSS polyimide composite films derived from mass loss.

Sample	SP‐ITO10‐UV	SP‐ITO50‐UV
Mass before exposure / mg	8.547 ± 0.022	11.245±0.036
Mass after exposure / mg	8.578 ± 0.022	11.236±0.018
Mass loss / mg	−0.031 ± 0.031	0.008±0.040
Erosion Yield / ×10^−24^ cm^3^ atom^−1^	−0.132 ± 0.133	0.034±0.168

**Table 8:** Surface atomic concentrations of ultrathin ITO/POSS polyimide composite films upon atomic oxygen exposure.

	Relative atomic concentration / %						
	C 1s	O 1s	Si 2p	N 1s	F 1s	In 3d	Sn 3d5
Unexposed SP‐ITO10‐UV	45.64	36.63	0.00	0.00	0.00	16.21	1.53
Exposed SP‐ITO10‐UV	42.97	38.37	0.00	0.00	0.00	17.13	1.53
Unexposed SP‐ITO20	42.83	37.98	0.00	0.00	0.00	16.47	1.72
Exposed SP‐ITO20	43.76	38.47	0.00	0.00	0.00	16.16	1.61
Unexposed SP‐ITO50‐UV	51.04	33.02	0.00	0.00	0.00	14.26	1.69
Exposed SP‐ITO50‐UV	37.49	42.52	0.00	0.00	0.00	17.90	2.10

**Table 9:** Sheet resistance of ultrathin ITO/POSS polyimide composite films upon atomic oxygen exposure.

	Sheet resistance / Ω □^−1^			
	Before AO exposure			After AO exposure
	Before bending	After 10 000 time bending (radius of 8 mm)	Before bending	After 10 000 time bending (radius of 8 mm)
Exposed SP‐ITO10‐UV	10 963.33±797.40	10 357.50±200.19	42 4600.00±20 6238.90	49 6266.70±11 2186.40
Exposed SP‐ITO20	454.49±79.45	432.88±92.26	1437.33±335.98	1410.42±269.31
Exposed SP‐ITO50‐UV	159.17±27.01	154.27±48.99	207.97±40.66	223.09±53.86

The transmittance and conductivity of ITO film (with 90% In_2_O_3_ and 10% SnO_2_) are related to the tradeoff of the oxygen vacancy in the material. A sufficient oxidation toward In_2_O_3_ and SnO_2_ is good for high transmittance, while a proper amount of oxygen vacancy is good for high conductivity (Figure 5e,f). To achieve high transmittance, a rich oxygen gas environment and a high substrate temperature up to 300 °C–400 °C are required. The conductivity of ITO mainly relies on the free electrons, which come from both oxygen vacancy and Sn doping. The oxygen vacancy releases some free electrons and increases the carrier concentration, for as ITO is neutral, an oxygen vacancy releases two free electrons. A doped Sn^4+^ take place of a In^3+^, and releasing a free electron. The ITO is a negative‐type semiconductor with free electron as the major carrier.

The ITO film in this work is sputtered using an ITO target at room temperature without additional oxygen gas. The existence of oxygen vacancy in SP‐ITO50‐UV offers more free electron carriers, which benefits a good conductivity (a low sheet resistance of 159.17 Ω □^−1^). After a 2.64 × 10^20^ O atoms cm^−2^ atomic oxygen exposure, some of the oxygen vacancies are filled, which benefits the transmittance increase in visible light range (400–800 nm) (Figure 5c). We also observe a transmittance decrease of ITO film in the infrared range, because the incident photon near infrared region is reflected due to the plasma vibration of the carriers in ITO.

For ITO, its carrier concentration is high (10^20^–20^21^ cm^−3^), while its carrier mobility is normal (10–30 cm^2^ V^−1^ s^−1^). The major carrier electrons in ITO come from both the oxygen vacancy and the Sn doping. As ITO is neutral, an oxygen vacancy releases two free electrons. When an oxygen vacancy is filled, two electrons are captured and confined inside the ionic bond of the indium oxide crystal. The quantitative relationship between carrier concentration and carrier mobility is given by Equation 8. To quantitively study the oxygen vacancy filling effect on carrier concentration and mobility, the measurements of σ, *n*, µ values of a standard control sample and a systematic experimental and theoretical study based on single variable is important. For example, the oxygen vacancy filling effects on σ values of different thickness ITO samples are different (Figure 5d). Along with oxygen vacancy filling, the carrier concentration *n* and conductivity σ decrease, while the carrier mobility µ would exhibit nonlinear relationship, and the transmittance would first increase and then decrease.

(8) σ=n·e·μ

where σ is the conductivity in S cm^−1^, *n* is the carrier concentration in cm^−3^, *e* = 1.6 × 10^−19^ C, µ is the carrier mobility in cm^2^ V^−1^ s^−1^.

### 2.6 Sealed Flexible Solar Cell and Predictive Model

#### 2.6.1 Atomic Oxygen Exposure to Sealed Flexible Solar Cell

**Figure 6:**
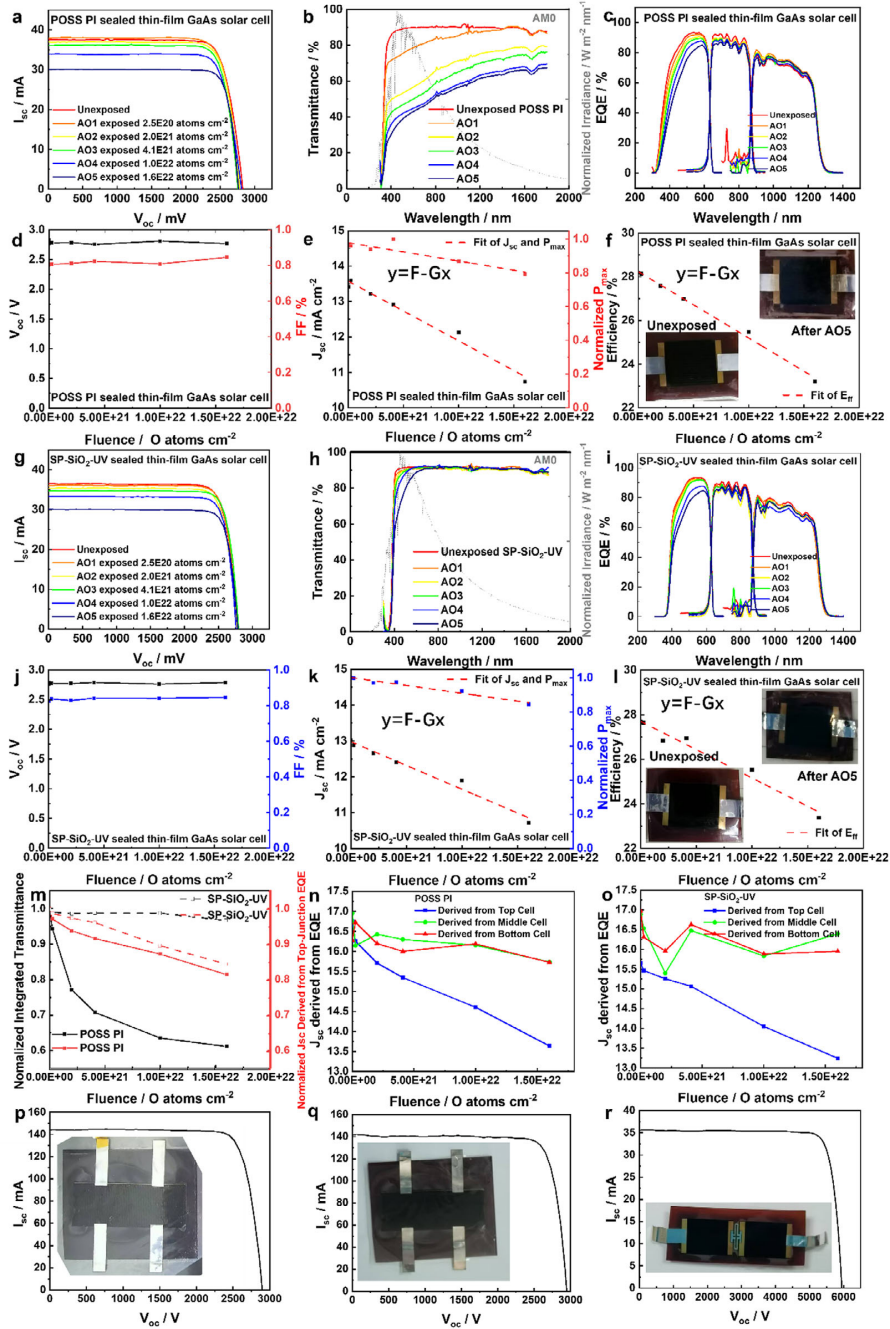
Performance of sealed triple‐junction GaAs thin‐film solar cells after atomic oxygen exposure. a) IV curve, b) transmittance of sealing film, c) EQE, d) *V*_oc_ and FF, e) *J*_sc_ and normalized power, f) efficiency of POSS polyimide sealed triple‐junction GaAs thin‐film solar cell. g) IV curve, h) transmittance of sealing film, i) EQE, j) *V*_oc_ and FF, k) *J*_sc_ and normalized power, l) efficiency of SP‐SiO_2_‐UV POSS polyimide sealed triple‐junction GaAs thin‐film solar cell. m) Normalized integrated transmittance and normalized *J*_sc_ derived from top‐cell EQE. *J*_sc_ derived from EQE of n) POSS polyimide sealed and o) SP‐SiO_2_‐UV POSS polyimide sealed triple‐junction GaAs thin‐film solar cells. IV curves of POSS polyimide sealed triple‐junction GaAs thin‐film solar cell p) before and q) after thermal cycling. r) IV curve of SP‐SiO_2_‐UV POSS polyimide triple‐junction GaAs thin‐film solar cell series.

An eight‐year long‐term atomic oxygen exposure in low Earth orbit (considering 2.0 × 10^21^ O atoms cm^−2^ year^−1^) has been conducted to the POSS polyimide, SP‐SiO_2_‐UV POSS polyimide films, and their sealed triple‐junction GaAs thin‐film solar cells, with five‐step increasing atomic oxygen fluences from 2.5 × 10^20^ (step 1, AO1), 2.0 × 10^21^ (step 2, AO2), 4.0 × 10^21^ (step 3, AO3), 1.0 × 10^22^ (step 4, AO4), to 1.6 × 10^22^ (step 5, AO5) O atoms cm^−2^ (**Figure** **6**; Tables S3 and S4, Supporting Information). For POSS polyimide film, the atomic oxygen erosion yield data are shown in Figure 2e. For SP‐SiO_2_‐UV POSS polyimide film, the atomic oxygen erosion yield data are 0.3 × 10^−25^ cm^3^ O atom^−1^ at 2.0 × 10^21^ O atoms cm^−2^, 0.1 × 10^−25^ cm^3^ O atom^−1^ at 4.0 × 10^21^ O atoms cm^−2^, 0.8 × 10^−26^ cm^3^ O atom^−1^ at 1.0 × 10^22^ O atoms cm^−2^, 0.6 × 10^−26^ cm^3^ O atom^−1^ at 1.6×10^22^ O atoms cm^−2^, which is one‐order of magnitude less than that of POSS polyimide, indicating an almost 0% mass loss of SP‐SiO_2_‐UV POSS polyimide after the long‐term atomic oxygen exposure. For both sealed solar cells, their *V*_oc_ values remain stable around 2.8 V upon the long‐term atomic oxygen exposure, with a 2.65% deviation of POSS polyimide sealed‐ and a 0.93% deviation of SP‐SiO_2_‐UV POSS polyimide sealed‐ solar cells (Figure 6a,d,g,j). The PL nondestructive testing to the sealed solar cells shows that the PL peak positions of the first‐ and third‐junctions of both sealed solar cells remain stable at around 630 and 1260 nm during the five‐step atomic oxygen exposure (Figure S9, Supporting Information). The stable *V*_oc_ values and PL peak positions indicate that there is no observed evidence for crystal defects at the depletion region of the top‐cell PN junction in the tandem‐junction solar cells.^[^ ^58^, ^59^ ^]^ When operating the PL steady‐state fluorescence spectroscope, the laser power is kept at 100%, and the output slits are adjusted when necessary, calibrating a control solar cell to reach a similar PL intensity. In spite of this, it is difficult to obtain accurate evaluations based on the deviation of peak intensity at the current state of art of the PL facility, especially for a series of experiments that are not detected at the same time. In light of this, the analysis of spectra usually focuses on the peak‐to‐peak intensity ratio and normalized peak intensity for multi‐peaks, and the peak position matters the most for single peak in this study. For solar cell semiconductor materials, the change in crystal directly and sensitively affects the bandgap and the PL peak position, resulting in the efficiency change.

The *J*_sc_ degradation of both solar cells is partially related to the transmittance decrease of the sealing films after the atomic oxygen exposure. The 5 eV atomic oxygen interaction depth in a polyimide is around 5 Å (Figure S10, Supporting Information), mainly causing a surface oxidation of the sealing film. The transmittance ($\int_{300\;nm}^{1800\;nm}Transmittance\;d\lambda$) of POSS polyimide decreases by 38.89% after the AO5 exposure (Figure 6b) because of the Rayleigh scattering of the formed porous SiO_x_ layer (Figure 1), while the transmittance of SP‐SiO_2_‐UV POSS polyimide film remained well, with only a 3.18% decrease after the AO5 exposure (Figure 6h; Table S5, Supporting Information). The atomic oxygen exposure mainly causes the top‐cell EQE ($\int_{300\;nm}^{700\;nm}EQE\;d\lambda$) degradation of both sealed solar cells, where POSS polyimide sealed solar cell exhibits a 22.34% degradation of after AO5, while the SP‐SiO_2_‐UV POSS polyimide sealed solar cell exhibits a 16.61% degradation (Figure 6c,i; Table S5, Supporting Information). The atomic oxygen exposure causes the *J*_sc_ degradation of both sealed solar cells, where POSS polyimide sealed solar cell exhibit a 19.97% *J*_sc_ degradation (with beginning of life (BOL) and end of life (EOL) *J*_sc_ of 13.42 and 10.74 mA cm^−2^) and a 17.67% efficiency degradation (with BOL and EOL efficiencies of 28.19% and 23.21%), while the SP‐SiO_2_‐UV POSS polyimide sealed solar cell exhibits a 17.73% *J*_sc_ degradation (with BOL and EOL *J*_sc_ of 13.03 and 10.72 mA cm^−2^) and a 15.50% efficiency degradation (with BOL and EOL efficiencies of 27.67% and 23.38%) (Figure 6e,f,k,l and Equations (9), (10); Table S6, Supporting Information). The *J*_sc_ degradation is mainly related to the top‐cell EQE degradation for tandem solar cells, where the EQE‐derived top‐cell *J*_sc_ degrades (Figure 6n,o and Equation 11). The SP‐SiO_2_‐UV POSS polyimide sealed solar cell exhibits better resistance to the eight‐year long‐term atomic oxygen exposure than the POSS polyimide, with 5.73% higher EQE, 2.17% higher *J*_sc_, and 2.24% higher efficiency (Tables S5,S6 and Figure S11, Supporting Information).

(9) $$FF=\frac{I_{mp}\;\times \;V_{mp}}{V_{oc}\;\times \;I_{sc}}$$

(10) $$E_{ff}=\;\;\frac{V_{oc}\times I_{sc}\times FF}{S\times Irradiance}=\frac{I_{mp}\times V_{mp}}{S\times Irradiance}$$

(11) $$J_{sc}=-q\int_{\lambda 1}^{\lambda 2}EQE\left(\lambda \right)\Phi_{ph,\lambda }^{AM0}d\lambda$$

where *FF* is the fill factor in %, *I_mp_* and *V_mp_* are the current and voltage at the maximum power point in A and V, *I_sc_* is short‐circuit current in A, *V_oc_* is open‐circuit voltage in V, *E_ff_* is the solar cell efficiency in %, *S* is the solar cell area in m^2^, *Irradiance* is the incident power density in W m^−2^, *J_sc_* is the short‐circuit current density in A m^−2^, *q* is the charge of electron 1.60 × 10^−19^ C, *EQE*(λ) is the external quantum efficiency, and $\Phi_{ph,\lambda }^{AM0}$ is the spectra photon flux in W m^−2^ nm^−1^ eV^−1^.

With the eight‐year long‐term atomic oxygen exposure, the transmittance conservation of SP‐SiO_2_‐UV POSS polyimide (transmittance loss of 3.18%) is significantly improved compared to POSS polyimide (transmittance loss of 38.89%). However, the improvement on the EQE, *J*_sc_, and efficiency of SP‐SiO_2_‐UV POSS polyimide sealed solar cell is less than expected, which is because the optical absorption coefficient α of GaAs‐based tandem solar cell material is really high (10^4^–10^5^ cm^−1^) in visible and near‐infrared range, and thus significantly reducing the film transmittance degradation effect on its sealed solar cell performance, and in this way the GaAs‐based solar cell gives generous tolerance to the transmittance degradation of surface sealing material. The SP‐SiO_2_‐UV POSS polyimide maintains high transmittance in solar spectrum after AO5 with a transmittance loss of 3.18%, however, its sealed solar cell still exhibits a 17.73% *J*_sc_ degradation, and a 15.50% efficiency degradation (Figure 6m). To explain this, a flexible glass (with 5% CeO_2_) sealed triple‐junction GaAs thin‐film solar cell is also exposed as a control sample, showing the same degradation trend, exhibiting a 13.33% *J*_sc_ degradation and a 9.50% efficiency degradation after 1.4 × 10^22^ O atoms cm^−2^ (a 7‐year atomic oxygen exposure in low Earth orbit). This is because of the co‐occurrence effect of ultraviolet of the atomic oxygen facility. Ultraviolet is produced accompanying with atomic oxygen in both laser‐detonation‐based and plasma‐neutralization‐based atomic oxygen facilities (Figure S12, Supporting Information). Ultraviolet causes the ionization effect on GaAs‐based solar cell, affecting the carriers and resulting in *J*_sc_ degradation, which is also evidenced by a previous study reporting an ultraviolet radiation effect on *J*_sc_ degradation on triple‐junction GaAs thin‐film solar cell.^[^ ^10^ ^]^ This is different from the silicon‐based solar cell, where both *V*_oc_ and *J*_sc_ degradations are observed after ultraviolet irradiation, which also indicates that the crystal structure of GaAs‐based semiconductor is more stable than silicon‐based semiconductor.^[^ ^60^, ^61^, ^62^, ^63^ ^]^

#### 2.6.2 Predictive Model and Mechanism

Atomic oxygen exposure produces linear degradation to *J*_sc_, *P*_max_, and *E*_ff_ of the sealed triple‐junction GaAs thin‐film solar cell performance as summarized in predictive Equation 12 (Figure 6e,f,k,l), which is different from electron or proton irradiation effects (exponential equation).^[^ ^64^, ^65^ ^]^

(12) y=F−G·x

where y is the final *J*_sc_ (*P*_max_, *E*_ff_) of the sealed solar cell in mA cm^−2^ (W, %), *F* is the initial *J*_sc_ (*P*_max_, *E*_ff_) of the sealed solar cell in mA cm^−2^ (W, %), *G* is the *J*_sc_ (*P*_max_, *E*_ff_) loss rate caused by an 1 cm^2^ of oxygen atoms in mA cm^−2^ (W, %)/O atoms cm^−2^, x is the atomic oxygen fluence in O atoms cm^−2^.

This is related to the reaction mechanism difference between atomic oxygen and electron/proton exposures. From the data and discussion above, the atomic oxygen and its accompanying ultraviolet induced top‐sealing‐film transmittance degradation are not the major reason for the cell performance degradation, for even the 0%‐transmittance‐degradation flexible glass sealed solar cell also exhibits the same performance degradation trend upon the long‐term atomic oxygen exposure. The 5 eV atomic oxygen generates a 5 Å penetration depth in polyimide, results in surface material oxidation (Figure S10, Supporting Information). The accompanying ultraviolet generates a ≈100 µm penetration depth in polyimide according to the Lambert's law, resulting in a bulk‐phase ionization effect to mainly to the top‐cell material according to the EQE data (Figure 6c,i). The surface oxidation does not affect the solar cell lattice, and the ultraviolet‐induced bulk‐phase ionization barely affects the GaAs‐based solar cell lattice (Figure S9, Supporting Information). The *V*_oc_ keeps stable and the J_sc_ decreases of the atomic oxygen and ultraviolet exposed GaAs‐based solar cells, which are observed by experiments both in this work and previous work.^[^ ^34^ ^]^ As the *V*_oc_ keeps stable during the long‐term atomic oxygen exposure, the depletion region of the top‐cell PN junction is unchanged, thus the interface evolution between the n‐type GaInP_2_ of the top cell and the antireflecting layer is considered (Figure S2, Supporting Information). The accompanying ultraviolet with high photon energy enables the excitation of electrons, the distortion of the electron cloud, the non‐stability of chemical bonds, resulting in more unsaturated bonds, dangling bond, and vacancy at the interface because of the lattice mismatch between two crystals, thus causing defects into interface and top‐cell semiconductor crystals, which affects the material's electronic structure and electrical property.^[^ ^25^, ^62^, ^63^ ^]^ The degradation mechanism is inferred to be related to interface traps, potentially P vacancies. The P defect formation energies of GaInP_2_ inside the crystal and on the surface were calculated to be 5.77 and 5.34 eV, respectively (Figure S13, Supporting Information).^[^ ^66^, ^67^ ^]^ As the unsaturated coordination of surface atoms, the chemical bonds on the surface are usually weaker than those inside the crystal. As the 200 nm photon energy is ≈6 eV, the ultraviolet photons have the potential to cause the P vacancy defects. The P vacancy exists at the interface between the top cell and the antireflecting layer, which is considered as the interface trap. At equilibrium, the probability for a trap to be filled with an electron is presented by the Fermi‐Dirac distribution (Equation 13).^[^ ^68^ ^]^ For a specific trap, the *f_te_* is a constant, and thus resulting in the linear degradation of J_sc_ along with time (Equation 12). The accompany ultraviolet photon with high energy benefits the bond breakage and photo‐oxidation. The bond breakage causes the chemical bond dissociation including the formation of P vacancy, and the photo‐oxidation causes the oxidation of top‐cell cations. The atomic oxygen mainly causes the surface oxidation, as it's an atmosphere, there might be some penetration into the interface between sealing film and the cell through the gap.

(13) $$f_{te}=\frac{1}{1+\exp\left(\frac{E_{t}-E_{f}}{kT}\right)}\;$$

where *f_te_* is the probability, *E_t_* − *E_f_* is the energy position of the trap with respect to the Fermi level, and *T* is the temperature.

Limited by the device's test depth, accuracy, and resolution, most experimentally interpretation of the atomic vacancy or elemental concentration requires the direct examining of the material surface, where thus relative studies focus on the investigation of solar cell materials (for example, ultraviolet effect on silicon solar cell material^[^ ^60^ ^]^), destructive testing based on focused ion beam technology or delicate material sample preparation for the deep level transient spectroscope. It is both an opportunity and a challenge to develop nondestructive testing technologies to experimentally investigate the evolution of atomic oxygen or ultraviolet effect on a polyimide sealed solar cell device, for it is constructive for in‐situ detecting the sealed device at practical working condition. As the polyimide sealed solar cell consists of a 50 µm thick polyimide film, a space silicone glue, and a solar cell, the XPS only detects the surface elements several nanometers deep (≈10 nm), and the ATR mode of FTIR only detects several micrometers deep (≈10 µm). There is a transmit mode of FTIR which requires the sample is transparent, while the bottom solar cell is almost black. In this study, we report a PL nondestructive testing technique for the sealed triple‐junction solar cells, which is good for detecting the in‐situ crystal structure of each layer (Figure S9, Supporting Information). As each layer has a different centered light absorption wavelength, this technique satisfies the requirements of nondestructive testing including test depth, accuracy, and resolution. However, the nondestructive testing technique of the element or chemical bond evolution for the sealed triple‐junction solar cells is still challenging, because of the undetectable depth and complex material system in each layer.

Combining with the stable PL peak position and *V*_oc_ value, the depletion region of the solar cell is well conserved. The interfacial defect induced trap center or recombination center is responsible for the degradation of *I*_sc_. The interfacial defect is a factor for the *I*_sc_ degradation, which is reported by other studies on solar cells.^[^ ^69^, ^70^ ^]^ From EQE data, the accompany ultraviolet induces the *I*_sc_ degradation of the top cell, which comes from the interfacial defects between the anti‐reflecting layer and the n‐type GaInP. There are some literatures on the P vacancy in GaInP based material.^[^ ^71^, ^72^ ^]^ From recent experimental observation data with the technology of high‐angle annular dark‐field scanning transmission electron microscopy on metal oxide and metal sulfide semiconductors, anion vacancy majors compared to cation vacancy, and one of the reasons is the anion has a smaller defect formation energy.^[^ ^73^, ^74^ ^]^ As GaAs‐based material is relatively expensive and is covalent bond dominant, there are few direct experimental observations on the elemental vacancy in GaAs based material yet and it is worth studying the irradiation effect on bare GaAs based solar cell or the solar cell materials in the future.

Flexible glass (most SiO_2_, containing 5% CeO_2_) is a commercial sealing material for thin‐film solar cell. Polyimide is with ultrasmall bending radius and more process feasibility such as film lamination or solution spraying. Table S7 (Supporting Information) lists the comparison of SP SiO_2_‐UV and flexible glass sealed thin‐film solar cells, in terms of AO erosion rate, transmittance retention rate, and efficiency attenuation rate (Table S7, Supporting Information). The mass and transmittance of flexible glass barely change upon atomic oxygen exposure. The SP SiO_2_‐UV film exhibits an ultrasmall erosion yield of 8 × 10^−27^ cm^3^ O atom^−1^ (close to a zero mass loss) and an ultrasmall transmittance drop of 1.1% in the range of 300–1800 nm at an atomic oxygen fluence of 1 × 10^22^ O atoms cm^−3^, which benefit its sealed solar cell efficiency conservation. The SP SiO_2_‐UV sealed thin‐film GaAs solar cell exhibit a 25.53% efficiency at an atomic oxygen fluence of 1 × 10^22^ O atoms cm^−3^, which is comparable to the flexible glass sealed thin‐film solar cell (a 25.83% efficiency at an atomic oxygen fluence of 1.4 × 10^22^ O atoms cm^−3^).

The POSS polyimide sealed triple‐junction GaAs thin‐film solar cell exhibits durable stability upon thermal cycling, with an *I*_sc_ of 141.4 mA, a *V*_oc_ of 2.94 V, an Eff of 29.18% before thermal cycling, and an *I*_sc_ of 140.9 mA, a *V*_oc_ of 2.95 V, an Eff of 29.27% after thermal cycling (Figure 6p,q). We first did a 44‐cycle thermal cycling without taking IV test data, where the adhesion of the POSS PI sealed solar cell is confirmed. We then did a 10‐cycle thermal cycling with IV test data before and after (Figure 6p,q). The initial several thermal cycling is important to verify the interfacial contact of an integrated device. Table S8 (Supporting Information) lists the summarize the thermal stability data of GaAs‐based solar cells from relevant publications.^[^ ^18^, ^19^, ^75^ ^]^ The materials in the integrated solar cell device are with good thermal stability, small thermal expansion coefficient, and good mechanical compatibility (rigid sealing material matching with rigid solar cell, flexible sealing material matching with flexible solar cell), and thus the solar cell performance keeps stable upon thermal cycling. Two flexible triple‐junction GaAs thin‐film solar cells are connected in series and sealed by SP‐SiO_2_‐UV POSS polyimide, exhibiting an *I*_sc_ of 35.62 mA, a *V*_oc_ of 5.95 V, and an *E*_ff_ of 29.72% (Figure 6r).

## 3 Conclusion

POSS polyimide composite has been designed to seal the triple‐junction GaAs thin‐film solar cell for long‐term low Earth orbit serve. Ultrathin oxide layer and ultraviolet absorbent have been introduced to improve the transmittance conservation and realize near zero atomic oxygen erosion yield. ITO/POSS polyimide has been designed for atomic oxygen durable conductive electrode. The endurance of POSS polyimide composite sealed triple‐junction GaAs thin‐film solar cell has been tested in terms of long‐term atomic oxygen (with BOL and EOL efficiencies of 27.67% and 23.38% at 1.60 × 10^22^ O atoms cm^−2^) and thermal cycling (with BOL and EOL efficiencies of 29.18% and 29.27% at 10 cycles of ±120 °C). The predictive equations for film erosion yield and sealed triple‐junction GaAs thin‐film solar cell upon the long‐term atomic oxygen exposure has been raised and explained in terms of zero‐/first‐order surficial chemical reactions of sealing film and the interface trap of the sealed solar cell. This study presents a practical way for space‐durable flexible photoelectronic devices in low Earth orbit, in terms of strategies, irradiations, mechanism, and predictive equations.

## 4 Experimental Section

### POSS Polyimide Composite

A 7.3 wt% Si_7_O_9_ POSS cage reinforced polyimide 50–70 µm thick is used as base (Figure S1, Supporting Information). 2 wt% of the ultraviolet absorbent 2‐(2H‐benzotriazol‐2‐yl)‐4,6‐di‐tert‐pentylphenol, CAS: 25973‐55‐1, Shanghai yuanye Bio‐Technology Co., Ltd) is dissolved in the POSS poly(amic acid). Sputtering and atomic layer deposition are applied for depositing various ultrathin oxide layers with thickness from 10 to 50 nm, including SiO_2_, Al_2_O_3_, ITO, with a sputtering deposition rate of 1.5 Å s^−1^.

### Triple‐Junction GaAs Thin‐Film Solar Cell

Triple‐junction GaAs thin‐film solar cell is sealed based on a sandwiched structure (Figure S2b, Supporting Information), with a top POSS polyimide composite sealing film, a middle triple‐junction GaInP_2_/GaAs/In_0.3_Ga_0.7_As thin‐film solar cell with coplanar electrodes, a bottom polyimide substrate, where space silicone glue is used between the interface of two layers. The IV test is conducted by an AM0 solar simulator from Spectrolab (SpectroSun X‐25 Mark II). The QE test is conducted by a quantum efficiency‐spectral response measurement system (Enlitech QE‐R3018). The photoluminescence spectroscopy (PL) test is conducted by a home‐built steady state and transient state fluorescence spectrometer (QM400‐TM2000).

### Atomic Oxygen and Ultraviolet Exposures

The hyperthermal neutral ground‐state atomic oxygen O(^3^P) beam is generated by a laser‐induced gas‐detonation facility ran at 2 Hz, with a yield of 91.6 at%, a velocity of 7.9 km s^−1^, a flux of 5.28 × 10^15^ O atoms cm^−2^ s^−1^, and a fluence of 2.64 × 10^20^ O atoms cm^−2^ (Figure S4a,b and Table S9, Supporting Information).^[^ ^76^, ^77^, ^78^, ^79^, ^80^, ^81^ ^]^ Round films are placed in the sample mount, strip films are taped on the sample mount, as labeled in Figure S4c (Supporting Information) and Table 1. Sample photographs before and after the atomic oxygen exposure are shown in Figure S4d,e (Supporting Information). The long‐term atomic oxygen exposure is conducted based on a microwave‐induced plasma and Mo reflector‐based neutralization system with a velocity of 7.9 km s^−1^ and a flux of 1.6 × 10^16^ O atoms cm^−2^ s^−1^, achieving a total fluence of 1.6 × 10^22^ O atoms cm^−2^.^[^ ^34^ ^]^ All samples are kept at 30 °C during the atomic oxygen exposures. Ultraviolet exposure is conducted to the films by a mercury lamp (Beijing NBET Technology Co. Ltd., LAB‐MERC1000) with 150 ESH. The mass is weighed by a semi‐micro balance (Ohaus, Model: EX125). The etch step is measured by a DekTak3 profiler (Veeco Instruments Inc.). The transmittance and reflectance of films are tested by a UV–vis–IR spectrometer (Varian, Lambda 950). Surface morphology is imaged by a field‐emission electron scanning microscope (FESEM, GeminiSEM 500). Surface chemistry is detected by an X‐ray photoelectron spectroscope (XPS, Thermo Fisher, ESCALAB 250Xi). The chemical bond information is tested by a Fourier transform infrared spectroscope (FTIR, Nicolet 6700). The error analysis in measurements is the uncertainties based on repeat experiments (n≥5).

### Thermal Cycling

The thermal cycling was conducted to the POSS polyimide sealed triple‐junction GaAs thin‐film solar cell for 10 cycles, from −120 to 120 °C, with a ramping rate of 10 °C min^−1^ and a holding time of 30 min at −120 and 120 °C. The total time for one cycle is 84 min, which is close to the actual working condition of 96 min cycle^−1^.

### Simulation and Calculation‐Finite Element Analysis Using COMSOL Software

**Model Construction**: Surface microstructures of the porous SiO_x_ grid were simulated using small particles with a length of 80 nm, height of 1000 nm, and spacing of 625 nm. The material was set to SiO_2_ with a real refractive index of 1.6. The external domain was configured as air with a real refractive index 1.0 and a width of 10 000 nm. Periodic boundary conditions were applied to the top and bottom boundaries. The left excitation port was positioned 10 000 nm from the left vertex of the particle.

**Transmittance and Reflectance (Frequency Domain) Analysis**: The right port was symmetrically placed 10 000 nm from the right base vertex of the particle. Perfectly Matched Layers (PMLs) with a thickness of 10 00 00 nm were added outside both ports to ensure accurate collection of transmitted and reflected energy.

**Frequency Domain Analysis**: Using the frequency domain module, a power of 1 W m^−1^ was injected at the left port, with slit conditions activated at both left and right ports. A parametric sweep from 200 to 2000 nm (step size: 1 nm) was performed to analyze the transmitted energy at both ports. These values were normalized as reflectance and transmittance, representing the reflected and transmitted energy of the microstructure, respectively.

**Dynamic Wave Propagation (Time Domain) Analysis**: To prevent reflected waves from the right port from interfering with results, the right port was positioned 40 µm from the left port. This ensured no light reached the right boundary within the simulation duration (0–100 fs). The minimum mesh size was set to 4.5 nm (in the computational domain), with a maximum mesh size of 48 nm, a maximum element growth rate of 1.3, and a curvature factor of 0.3. Based on the geometric characteristics of the regions, manual meshing was implemented to maintain both mesh symmetry and density during optical simulations.

**Time Domain Analysis**: The time domain module was employed to solve transient solutions for electromagnetic waves at 480 nm and 1000 nm wavelengths within 0–100 fs (step size: 0.1 fs). The electric field was configured as a sinusoidal traveling wave (Equation 14).

(14) $$E_{d}=A\sin\left(2\pi \frac{c}{\lambda }\times t\right)$$

where *E*_d_ is the confined electric filed in V m^−1^, *A* is the electric field strength (set to 100 V m^−1^), *c* is the speed of light, *λ* is the wavelength, and *t* denotes time.

### Molecular Dynamics (MD) and Density Functional Theory (DFT) Calculations

Atomic oxygen bombardment dynamics were simulated using CP2K.^[^ ^82^ ^]^ Input files were generated with Multiwfn, and MD simulations employed the GFN‐xTB method.^[^ ^83^, ^84^ ^]^ The environmental temperature was set to 298.15 K using the Canonical Sampling through Velocity Rescaling (CSVR) thermostat. The model's bottom layer was fixed. Atomic oxygen bombardment energy was set to 5 eV, with an initial velocity of 0.0036 a.u. (atomic units). The low‐Earth orbit atomic oxygen flux of ≈10^13^–10^16^ atoms cm^−2^ s^−1^ was approximated as ≈0.1 atom Å^−2^ s^−1^. Atomic oxygen counts were determined based on cross‐sectional areas and a density of ≈0.1 atom Å^−2^, rounded to the nearest integer array dimensions: Polyimide cross‐section is 20.17 × 15.78 Å^2^ and rounded to 8 × 4 oxygen atom array, SiO_2_ cross‐section is 18.63 × 18.63 Å^2^ and rounded to 6 × 6 array, Al_2_O_3_ cross‐section is 19.22 × 19.22 Å^2^ and rounded to 7 × 6 array. The defect formation energy was calculated using the PBE functional.

Quantum Espresso calculated the band structures of In_2_O_3_ with/without oxygen vacancies. SeeK‐path generated high‐symmetry k‐point paths, VESTA rendered atomic images, while Jmol, VMD, and VideoMach produced animations.^[^ ^85^, ^86^, ^87^, ^88^, ^89^, ^90^ ^]^

## Conflict of Interest

The authors declare no conflict of interest.

## Author Contributions

M.Q. and M.W. contributed equally to this work.

## Supporting information



Supporting Information

Supplemental Video 1

Supplemental Video 2

Supplemental Video 3

Supplemental Video 4

Supplemental Video 5

## Data Availability

The data that support the findings of this study are available from the corresponding author upon reasonable request.
