# Supplementary material for: POSS Polyimide Composite Sealed Triple‐Junction GaAs Thin‐Film Solar Cell for Long‐Term Low Earth Orbit Serve
Source: Adv Sci (Weinh). 2025 Dec 15;13(12):e16383. doi: 10.1002/advs.202516383 (PMC12948234; doi:10.1002/advs.202516383)
Supplement: Supplementary file 1 — Supporting Information [file ADVS-13-e16383-s003.docx]

***Supporting Information for***

**POSS Polyimide Composite Sealed Triple-Junction GaAs Thin-Film Solar Cell for Long-Term Low Earth Orbit Serve**

Min Qian^1^[[1]](#footnote-1)^*^, Min Wu^2*^, Xiaoyang Xuan^3*^, Yang Gao^4^

*^1^ School of Physics, East China University of Science and Technology, Shanghai 200237, People's Republic of China*

*^2^ State Key Laboratory of Space Power-sources Technology, Shanghai Institute of Space Power-Sources, Shanghai 200245, People’s Republic of China*

*^3^* *College of Chemistry and Chemical Engineering, Taishan University, Taian, Shandong 271000, People's Republic of China*

*^4^ School of Mechanical and Power Engineering*

*Shanghai Key Laboratory of Intelligent Sensing and Detection Technology*

*East China University of Science and Technology*

*Shanghai 200237, People’s Republic of China*


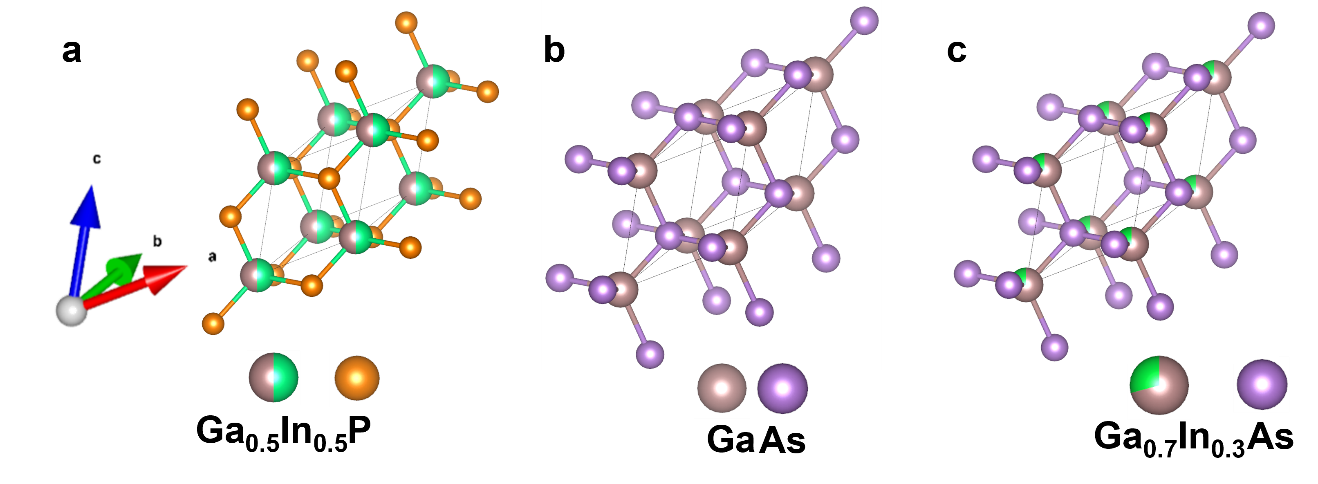


Figure S1. Crystal structures of GaInP_2_, GaAs, and In_0.3_Ga_0.7_As.


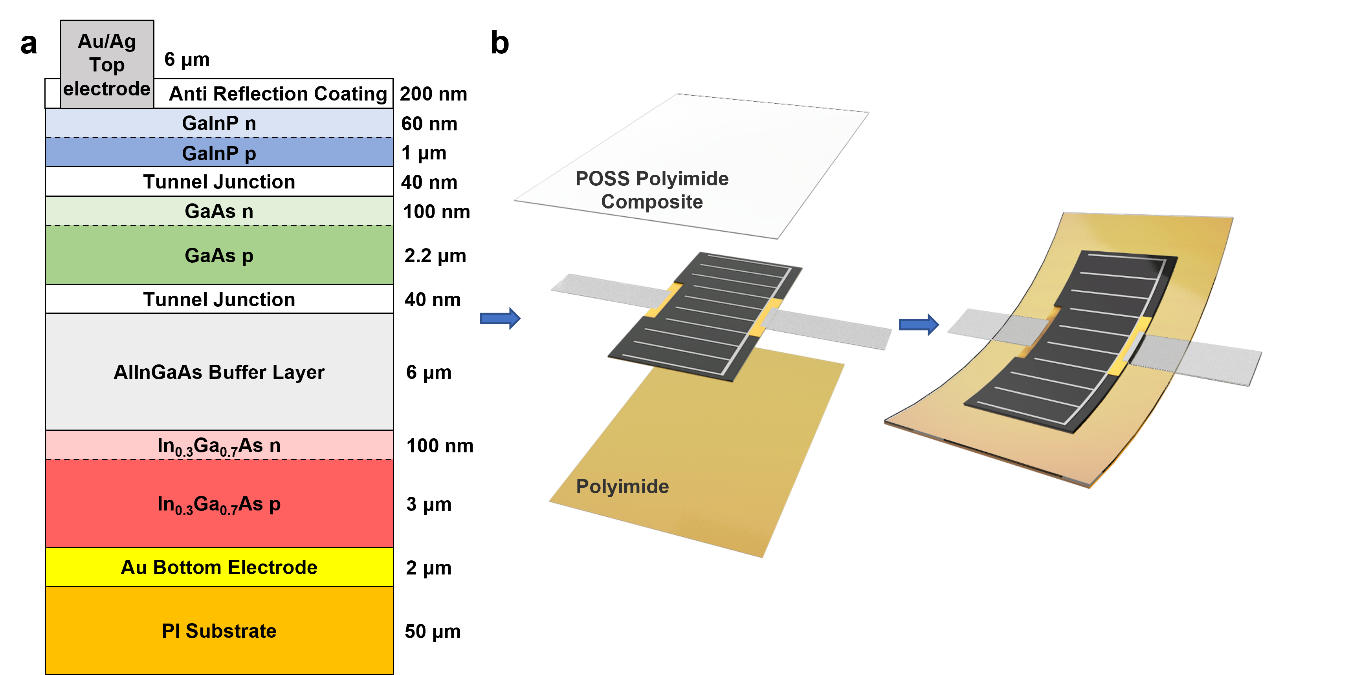


Figure S2. Cell structure of triple-junction GaAs thin-film solar cell.

Table S1. Bonding energy in polyimide.

| Chemical bond | Bonding energy /kJ mol^-1^ | Bonding energy / eV |
| --- | --- | --- |
| C-C | 332 | 3.45 |
| C-H | 414 | 4.31 |
| C-O | 326 | 3.39 |
| C-N | 305 | 3.17 |
| C-F | 485 | 5.04 |
| C=C | 611 | 6.35 |
| C=O | 728 | 7.57 |

Table S2. FTIR spectra assignments of POSS polyimide films.

| **Wavenumber / cm^-1^** | **Functional Group** |
| --- | --- |
| 665-910 | Benzene ring |
| 1005 | δ_CH_ |
| 1040-1050 | ν_C-O_ |
| 1125 | ν_C-N_ |
| 1237 | ν_C-N_ |
| 1257 | ν_C-O_ |
| 1320 | δ_CH3_ |
| 1376 | δ_CH3_ |
| 1435, 1485 | Benzene ring |
| 1620 | ν_C=C_ |
| 1713 | ν_C=O_ |
| 1780 | ν_C=O_ |
| 2965 | ν_CH3_ |
| 3074 | ν_C-H_ |
| 3500 | νo_-H,_ ν_N-H_ |


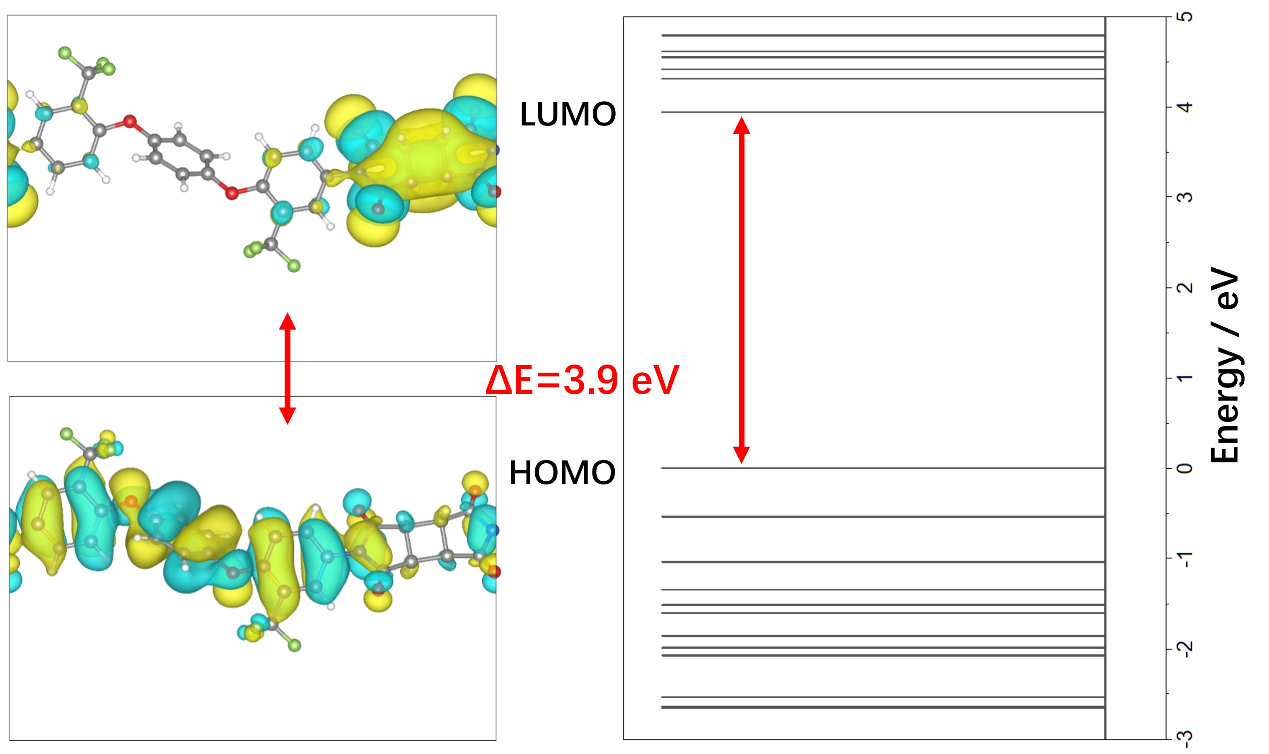


Figure S3. HOMO-LUMO structure of polyimide.


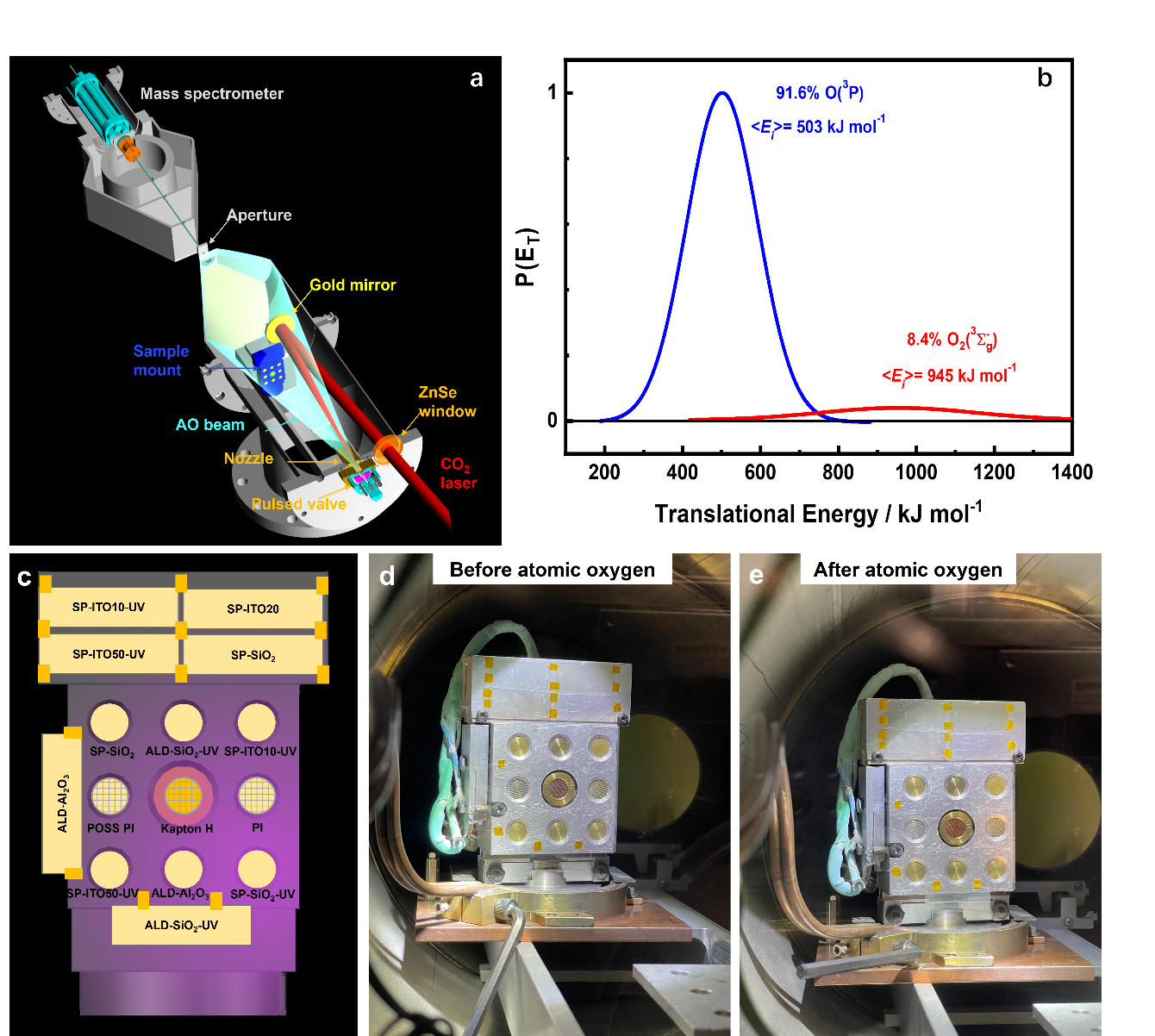


Figure S4. Atomic oxygen exposure. (a) Setup, and (b) translational energy of the hyperthermal atomic oxygen beam. (c) Film sample position, and sample photographs (d) before and (e) after the 2.64×10^20^ O atoms cm^-2^ atomic oxygen exposure.


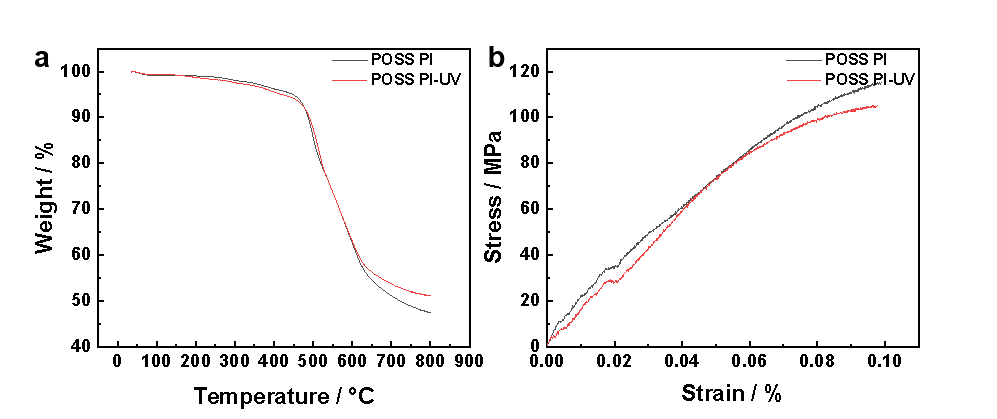


Figure S5. (a) Thermogravimetric analysis and (b) mechanical tensile strength of POSS polyimides with and without ultraviolet absorbent.


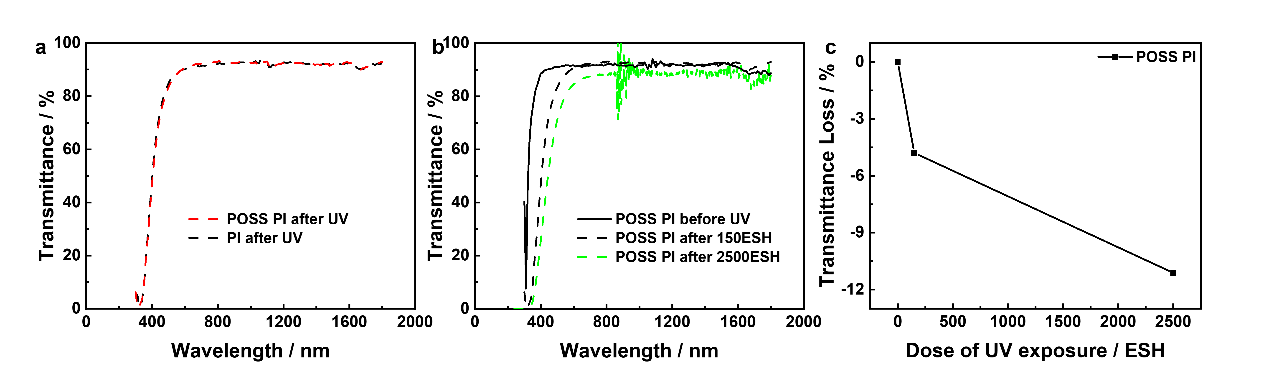


Figure S6. (a) Transmittance of POSS polyimide and polyimide films after 150 ESH ultraviolet exposure. (b) Transmittance and (c) transmittance loss of POSS polyimide films after 150 and 2500 ESH ultraviolet exposure.


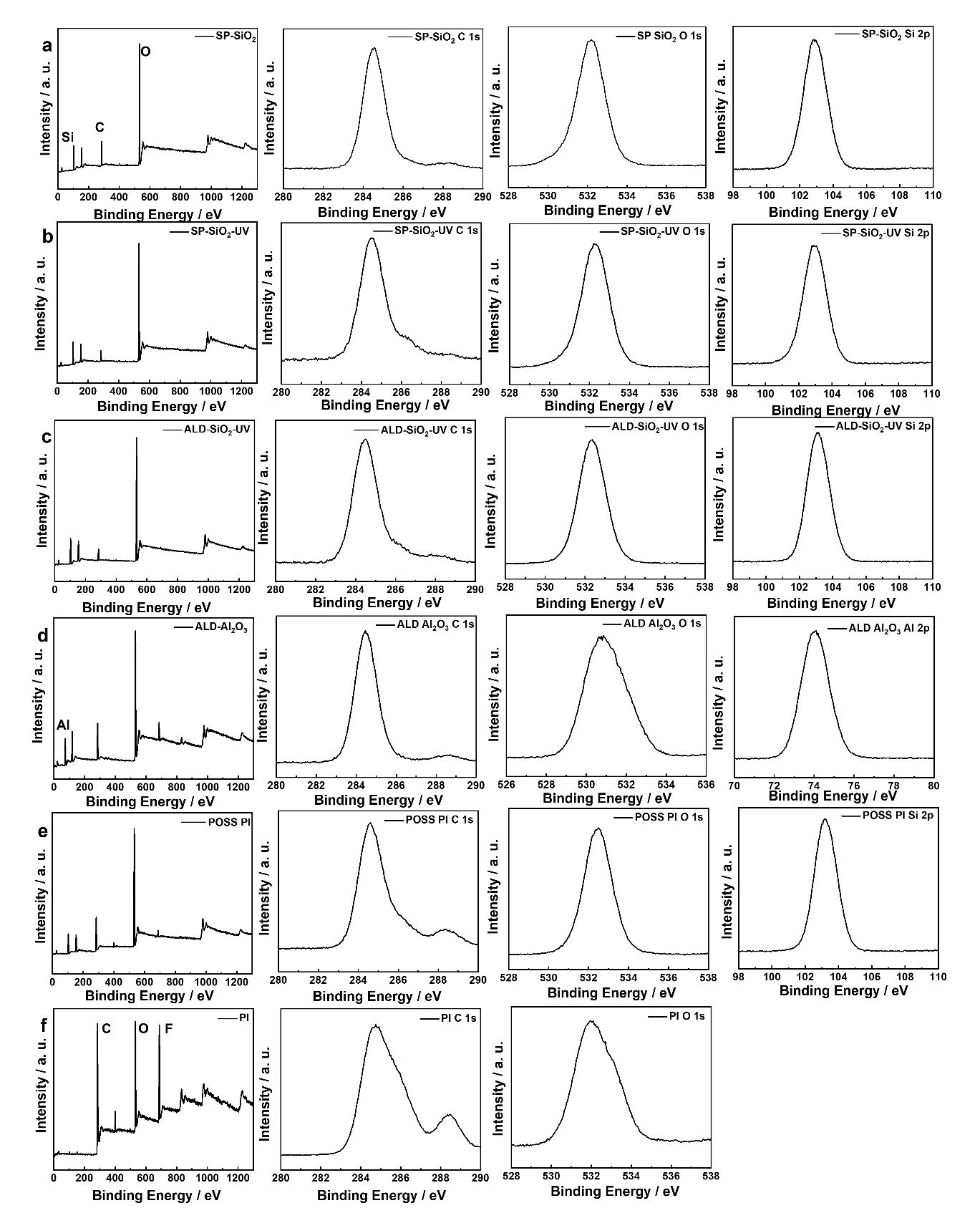


Figure S7. Surface Chemistry. XPS spectrum of (a) SP-SiO_2_, (b) SP-SiO_2_-UV, (c) ALD-SiO_2_-UV, (d) ALD-Al_2_O_3_, (e) POSS PI, (f) PI, after the 2.64×10^20^ O atoms cm^-2^ atomic oxygen exposure.


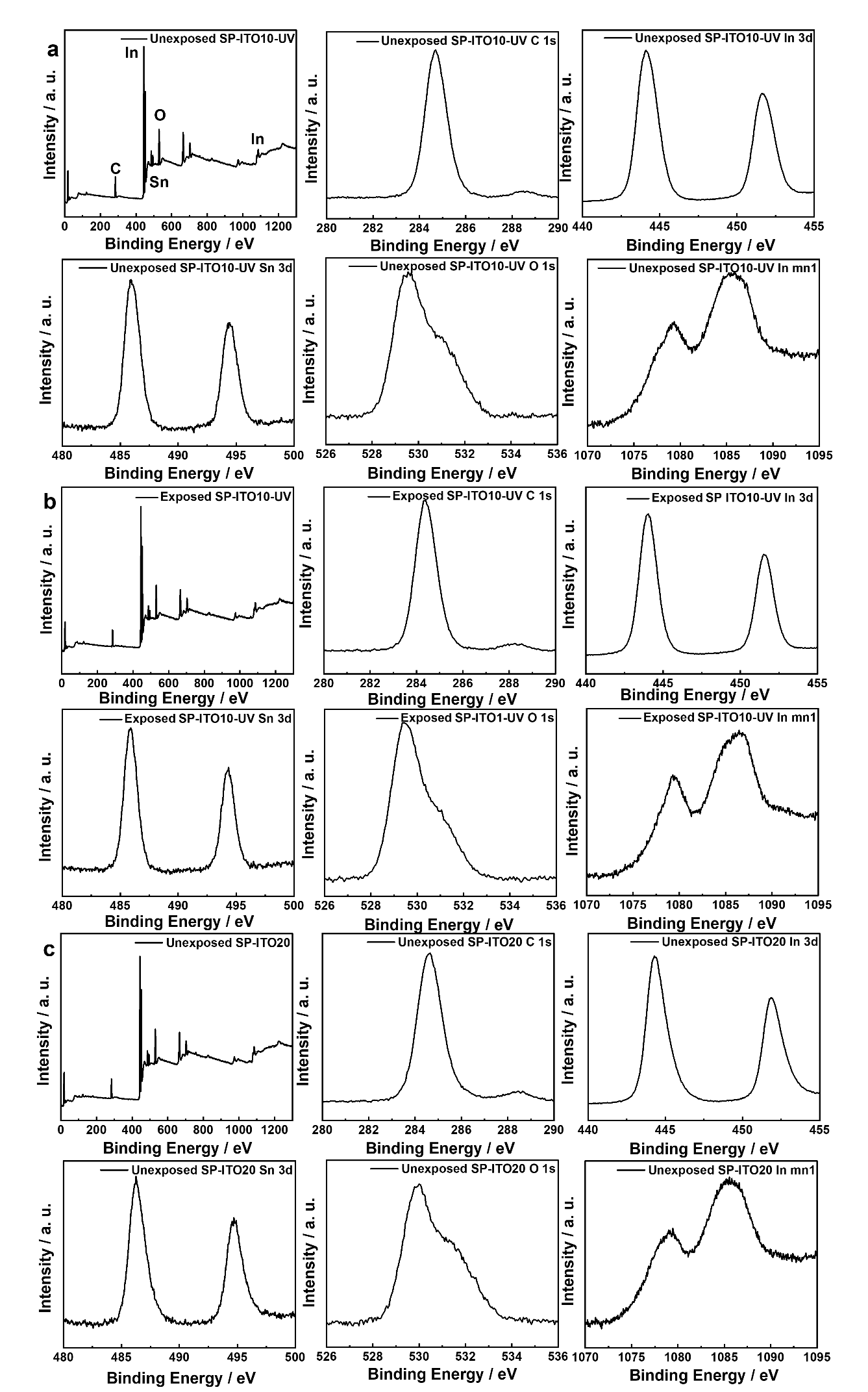


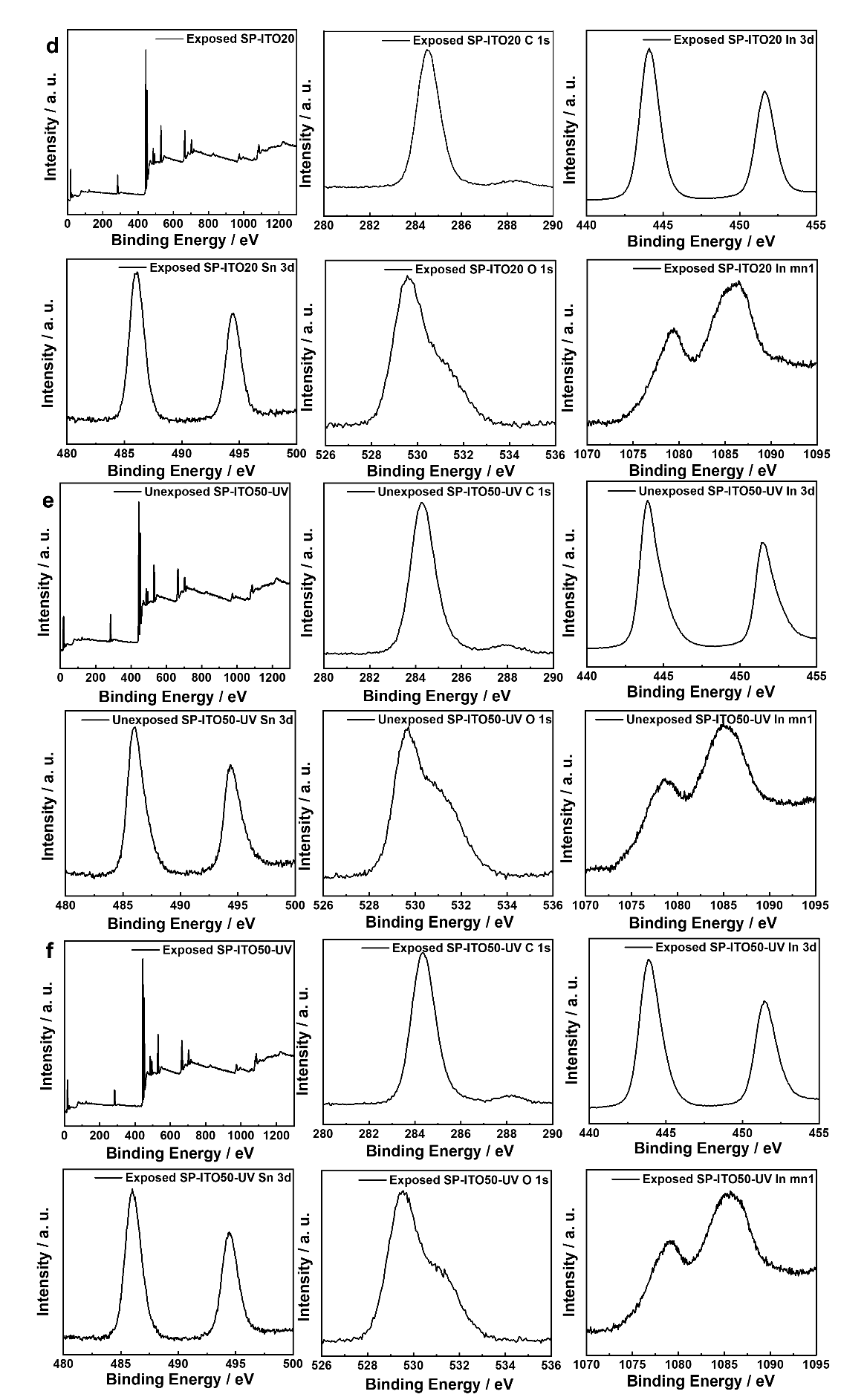


Figure S8. Surface chemistry. XPS spectrum of (a,b) SP-ITO10-UV, (c,d) SP-ITO20, (e,f) SP-ITO50-UV, before and after the 2.64×10^20^ O atoms cm^-2^ atomic oxygen exposure.

Table S3. Data of POSS polyimide sealed triple-junction GaAs thin-film solar cell before and after the five-step 2.50 × 10^20^ (AO1), 2.0 × 10^21^ (AO2), 4.0 × 10^21^ (AO3), 1.0 × 10^22^ (AO4), and 1.6 × 10^22^ (AO5) atoms cm^-2^ AO exposure.

|  | Area  / cm^2^ | Jsc  / mA cm^-2^ | Voc  / V | FF  / % | Eff  / % | Rss  / Ohm | Rshs  / Ohm |
| --- | --- | --- | --- | --- | --- | --- | --- |
| Before AO | 2.8 | 13.42 | 2.827 | 80.4 | 28.19 | 5.717 | 5657.7 |
| After AO1 | 2.8 | 13.60 | 2.779 | 80.5 | 28.14 | 0.803 | 26405 |
| After AO2 | 2.8 | 13.22 | 2.784 | 81.1 | 27.58 | 5.627 | 4189.9 |
| After AO3 | 2.8 | 12.90 | 2.752 | 82.3 | 26.98 | 3.049 | 19805 |
| After AO4 | 2.8 | 12.12 | 2.810 | 80.9 | 25.47 | 7.184 | / |
| After AO5 | 2.8 | 10.74 | 2.766 | 84.5 | 23.21 | 5.034 | 4693.1 |

Table S4. Data of SP-SiO_2_-UV POSS polyimide sealed triple-junction GaAs thin-film solar cell before and after the five-step 2.50 × 10^20^ (AO1), 2.0 × 10^21^ (AO2), 4.0 × 10^21^ (AO3), 1.0 × 10^22^ (AO4), and 1.6 × 10^22^ (AO5) atoms cm^-2^ AO exposure.

|  | Area  / cm^2^ | Jsc  / mA cm^-2^ | Voc  / V | FF  / % | Eff  / % | Rss  / Ohm | Rshs  / Ohm |
| --- | --- | --- | --- | --- | --- | --- | --- |
| Before AO | 2.8 | 13.03 | 2.787 | 82.5 | 27.67 | 4.203 | 79210 |
| After AO1 | 2.8 | 12.88 | 2.773 | 83.8 | 27.64 | 2.488 | 3233.1 |
| After AO2 | 2.8 | 12.65 | 2.773 | 82.8 | 26.83 | 4.310 | 10614.3 |
| After AO3 | 2.8 | 12.40 | 2.790 | 84.2 | 26.94 | 3.111 | 14403.2 |
| After AO4 | 2.8 | 11.90 | 2.763 | 84.1 | 25.53 | 4.444 | 14505.5 |
| After AO5 | 2.8 | 10.72 | 2.789 | 84.6 | 23.38 | 4.893 | 9971.9 |


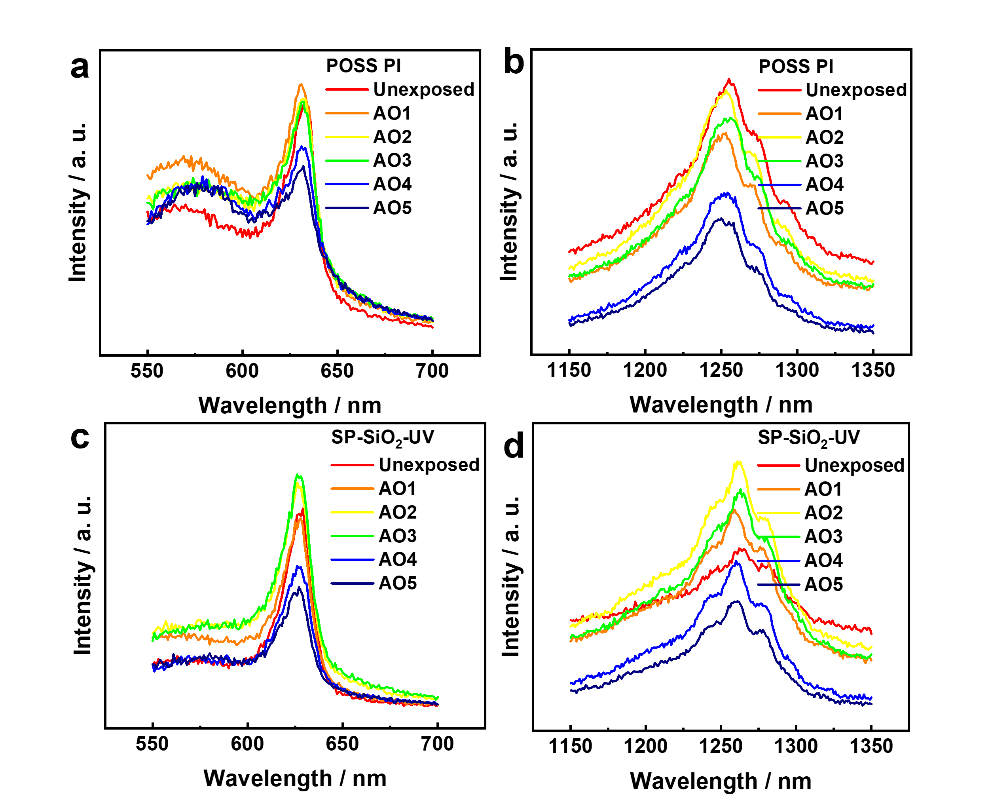


Figure S9. PL spectra of (a) the top junction (λ_ex_=532 nm), and (b) the bottom junction (λ_ex_=1040 nm) of the POSS PI sealed triple-junction GaAs thin-film solar cell. (c) PL spectra of (a) the top junction (λ_ex_=532 nm), and (b) the bottom junction (λ_ex_=1040 nm) of the SP-SiO_2_-UV sealed triple-junction GaAs thin-film solar cell.


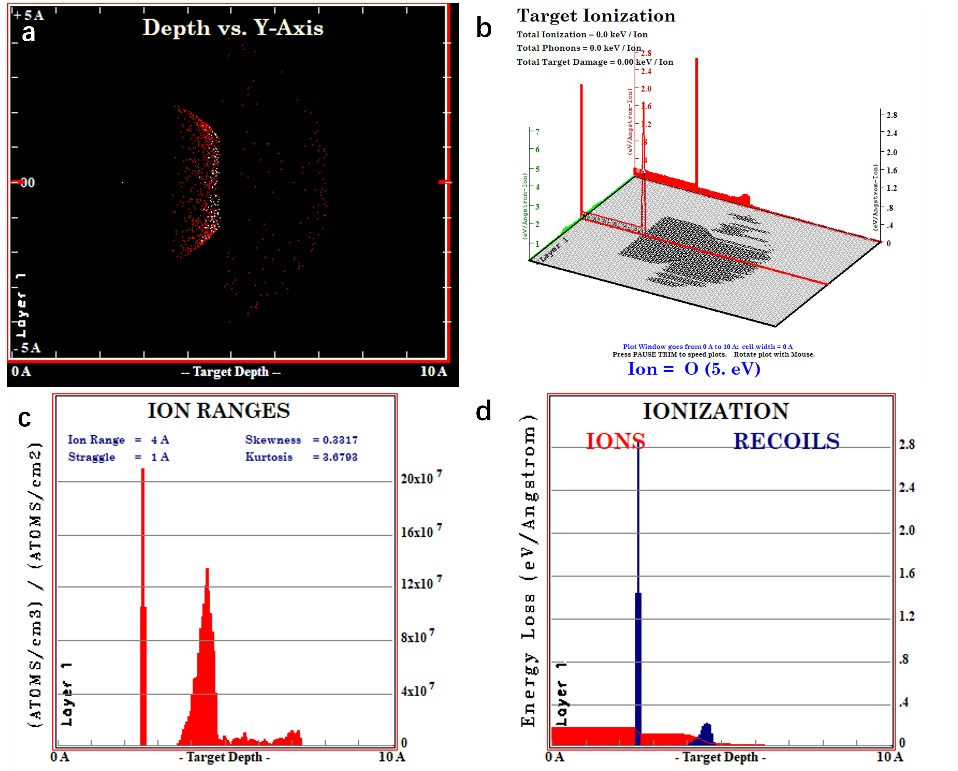


Figure S10. (a) Spatial distribution, (b) energy loss, (c) particle distribution, (d) ionization loss of 5 eV atomic oxygen in polyimide.

Table S5. Atomic oxygen effects on film transmittance and the top-junction EQE of sealed triple-junction GaAs thin-film solar cells.

| Atomic oxygen fluence | Film Transmittance | | | Sealed Solar Cell | |
| --- | --- | --- | --- | --- | --- |
|  | POSS PI | SP-SiO_2_-UV | POSS PI | | SP-SiO_2_-UV |
|  | $\int_{300nm}^{1800nm} Td\lambda$ | $\int_{300nm}^{1800nm} Td\lambda$ | $\int_{300nm}^{700nm} EQEd\lambda$ | | $\int_{300nm}^{700nm} EQEd\lambda$ |
| Before AO | 130903 | 129324 | 23604 | | 20895 |
| AO1 | 123479 | 128249 | 22674 | | 20629 |
| AO2 | 100919 | 127700 | 21714 | | 20298 |
| AO3 | 925567 | 127938 | 21059 | | 19988 |
| AO4 | 83122 | 127849 | 19815 | | 18543 |
| AO5 | 79986 | 125206 | 18331 | | 17424 |

Table S6. Atomic oxygen effects on the sealed triple-junction GaAs thin-film solar cell performance.

| Atomic oxygen fluence |  | Sealed Solar Cell | | | | | |  |
| --- | --- | --- | --- | --- | --- | --- | --- | --- |
|  | POSS PI | | | | SP-SiO_2_-UV | | |  |
|  | V_oc_  / V | | J_sc_  / mA cm^-2^ | Efficiency / % | V_oc_  / V | J_sc_  / mA cm^-2^ | Efficiency / % | |
| Before AO | 2.827 | | 13.42 | 28.19 | 2.787 | 13.03 | 27.67 | |
| AO1 | 2.779 | | 13.60 | 28.14 | 2.773 | 12.88 | 27.64 | |
| AO2 | 2.784 | | 13.22 | 27.58 | 2.773 | 12.65 | 26.83 | |
| AO3 | 2.752 | | 12.90 | 26.98 | 2.79 | 12.40 | 26.94 | |
| AO4 | 2.81 | | 12.12 | 25.47 | 2.763 | 11.90 | 25.53 | |
| AO5 | 2.766 | | 10.74 | 23.21 | 2.789 | 10.72 | 23.38 | |


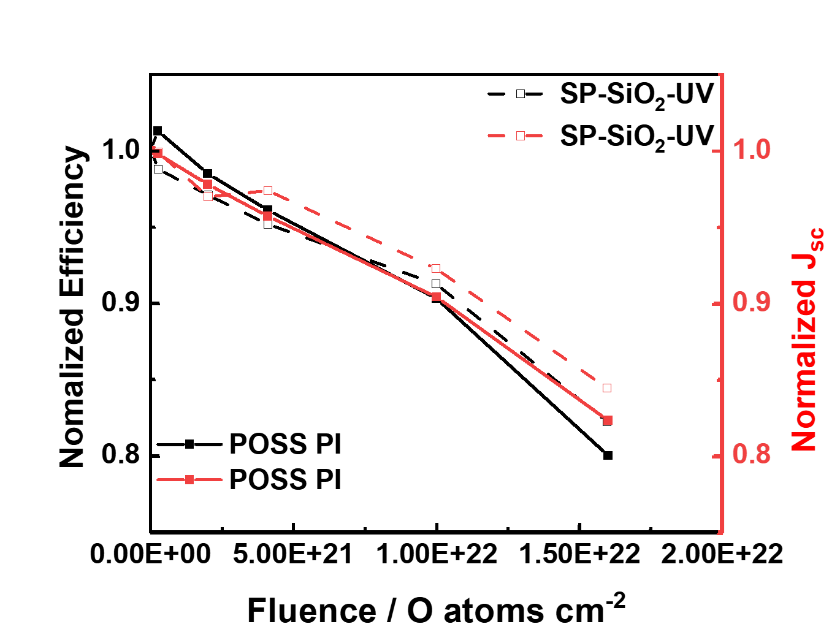


Figure S11. Normalized efficiency and J_sc_ data of POSS PI and SP-SiO_2_-UV films sealed triple-junction GaAs thin-film solar cells.


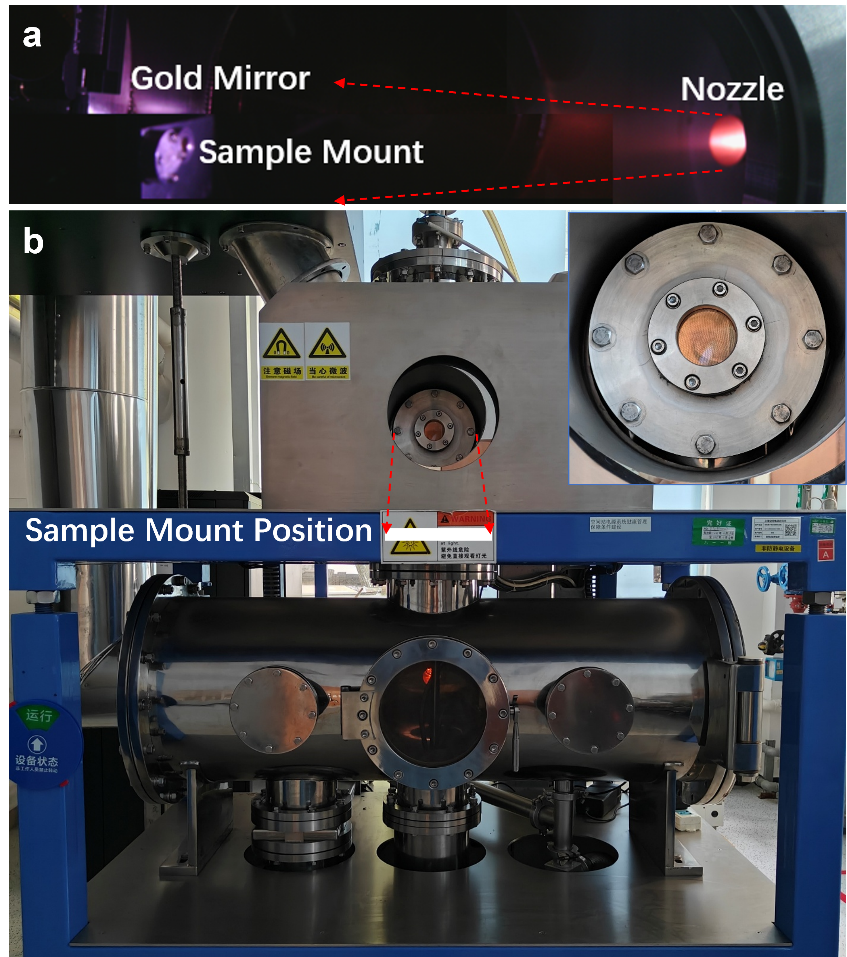


Figure S12. The co-occurrence effect of ultraviolet of the atomic oxygen facility. Photographs of ultraviolet produced accompanying with atomic oxygen in (a) laser-detonation-based and (b) plasma-neutralization-based atomic oxygen facilities.


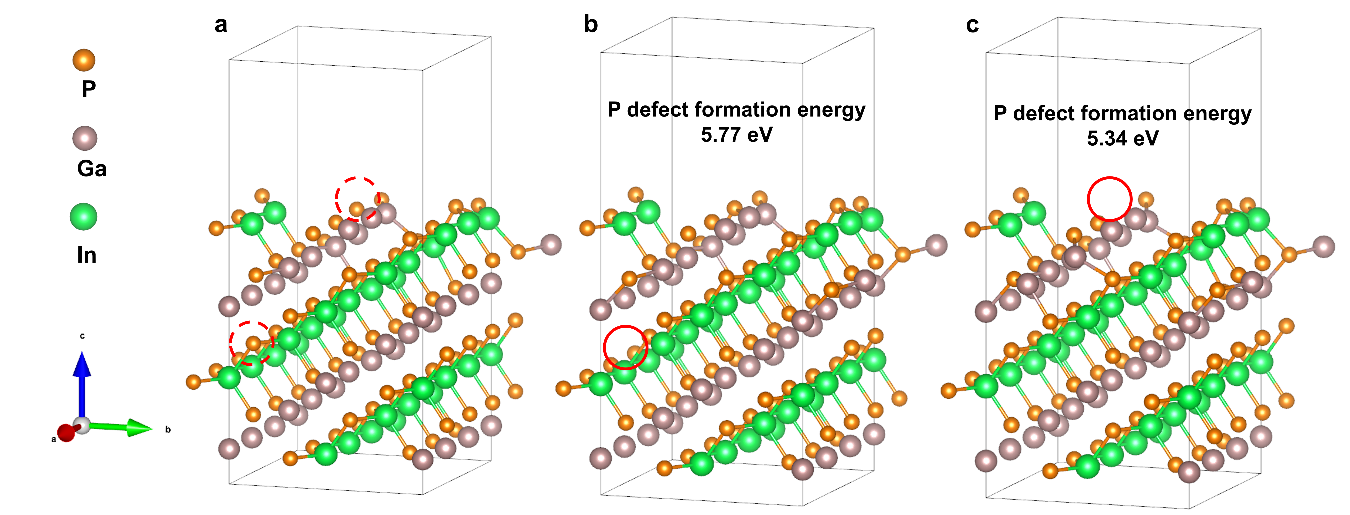


Figure S13. The P vacancy formation energy in GaInP. (a) P atoms inside the crystal and on the surface. (b) P defect formation energy inside the crystal. (c) P defect formation energy on the surface.

Table S7. Comparison of SP SiO_2_-UV and flexible glass sealed thin-film solar cells.

|  | Erosion Yield / ×10^-24^ cm^3^ O atom^-1^ | | Efficiency of Sealed Solar Cell / % | | Transmittance Loss (300-1800 nm) / % | |
| --- | --- | --- | --- | --- | --- | --- |
|  | SP-SiO_2_-UV | Flexible Glass | SP-SiO_2_-UV | Flexible Glass | SP-SiO_2_-UV | Flexible Glass |
| Before AO | 0 | NA | 27.67 | 27.63 | 0 | NA |
| After AO | 0.008  (1×10^22^ O atoms cm^-3^) | NA | 25.53  (1×10^22^ O atoms cm^-3^) | 25.83  (1.4×10^22^ O atoms cm^-3^) | 1.1%  (1×10^22^ O atoms cm^-3^) | NA |

Table S8. Thermal stability data of GaAs-based solar cells from this work and relevant publications.

| Sample | Temperature Range | Cycle times | Efficiency Loss / % | Reference |
| --- | --- | --- | --- | --- |
| Unsealed GaInP/GaAs/InGaAs  (Flexible solar cell) | -60~75 ^o^C | 420 | <2% | [19] |
| Anti-radiation glass/GaAs (Rigid solar cell) | -80~160 ^o^C | 600 | 0 | [75] |
| Pseudomorphic glass/GaInP/InGaAs/Ge/PI (Flexible solar cell) | -150~150 ^o^C | 2000 | 0 | [18] |
| POSS PI composite/ GaInP/GaAs/InGaAs/PI (Flexible solar cell) | -120~120 ^o^C | 10 | 0 | This work |

S1. Preparation of POSS polyimide

A 100 mmol (4-bis(4-amino-2-trifluoromethylphenoxy)benzene were put in a three-neck flask with 300 g DMAc solvent, stirring in an ice-water bath for 30 min with nitrogen flow. A100 mmol 1,2,3,4-cyclobutanetetracarboxylic dianhydride and 50 g DMAc solvent were added, stirring in the ice-water bath for 2 h. The solution was further stirred for 24 h at room temperature to form a poly(amic acid) solution with a 15 wt% solid constant. A 16.76 mmol POSS (CAS No. 444315-26-8) was added into the poly(amic acid) solution and stirred for 30 min. The POSS poly(amic acid) solution was spin-coated onto a glass substrate at 1000 rpm for 30 sec and set in a dry cabinet for two days to evaporate residue DMAc solvent, and then was put in a vacuum oven for curing at 50 ^o^C for 30 min, 100 ^o^C for 30 min, 200 ^o^C for 30 min, and 270 ^o^C for 20 min. The obtained transparent POSS reinforced polyimide ~50 μm thick was peeled off glass.

Table S9. Flux and fluence of the atomic oxygen beam.

| Sample | Shots | Hz | Fluence /  O atoms cm^-2^ | Flux /  O atoms cm^-2^ sec^-1^ | Kapton-H Reference Sample Erosion Depth / µm |
| --- | --- | --- | --- | --- | --- |
| Kapton H | 100,000 | 2 | 2.64±0.05×10^20^ | 5.28±0.09×10^15^ | 7.92±0.14 |

1. ** Corresponding Authors. Min Qian. E-mail:* [*mqian@ecust.edu.cn*](mailto:mqian@ecust.edu.cn)

   *Min Wu. E-mail:* [*minwindyw@163.com*](mailto:minwindyw@163.com)

   *Xiaoyang Xuan. E-mail: xyxuan@tsu.edu.cn*  [↑](#footnote-ref-1)
